# Dynamic prognostic prediction in sepsis using longitudinal blood gas trajectories: development and external validation

**DOI:** 10.3389/fmed.2026.1852841

**Published:** 2026-06-12

**Authors:** Le-run Zong, Qian-yu Bi, Yang Liu, Shu-jiao Lu, Cun-yang Li, Ze-kun Wei, Te-jin Ba, Li Kong, Fei-hu Zhang

**Affiliations:** 1 Shandong University of Traditional Chinese Medicine, Jinan, China; 2 Department of Emergency Center, Affiliated Hospital of Shandong University of Traditional Chinese Medicine, Jinan, China; 3 Department of Emergency and Critical Care Medicine, International Mongolian Medical Hospital of Inner Mongolia Autonomous Region, Hohhot, China

**Keywords:** arterial blood gas, dynamic prediction, growth mixture model, in-hospital mortality, sepsis, trajectory analysis

## Abstract

**Objective:**

To develop and externally validate a dynamic prognostic framework for sepsis based on longitudinal arterial blood gas trajectories, and to assess whether ABG-derived dynamic features provide incremental prognostic information beyond conventional clinical variables.

**Methods:**

This retrospective observational study used MIMIC-IV for model development and internal validation, and eICU-CRD for independent external validation. Adult ICU patients with sepsis and sufficient repeated ABG measurements within 168 h were included. Eight ABG-related variables were analyzed: PaO_2_, PaCO_2_, FiO_2_, PaO_2_/FiO_2_ ratio, pH, base excess, lactate, and glucose, summarized in 12-h windows. Latent class growth analysis and growth mixture modeling identified longitudinal ABG trajectories in MIMIC-IV, and the locked models were directly applied to eICU-CRD. Dynamic prediction models were developed using data accrued up to days 3, 5, and 7 with elastic-net logistic regression. Performance was evaluated by ROC-AUC, PR-AUC, Brier score, calibration, and decision curve analysis.

**Results:**

After applying the longitudinal ABG completeness criteria, 1,378 patients from MIMIC-IV and 647 from the eICU-CRD external validation cohort were included. Clinically interpretable trajectories were identified for all eight ABG variables, suggesting dynamic physiological heterogeneity in sepsis. For post-landmark in-hospital mortality prediction, models integrating clinical variables, ABG trajectory posterior probabilities, and ABG summary statistics showed moderate discrimination. In MIMIC-IV internal validation, ROC-AUCs using data accrued up to days 3, 5, and 7 were 0.792, 0.796, and 0.808, respectively; the corresponding values in eICU-CRD external validation were 0.658, 0.679, and 0.703. Compared with the clinical reference model, adding ABG-derived dynamic features yielded small-to-moderate improvements in external validation, with ROC-AUC increases of 0.036, 0.044, and 0.055 across the three landmarks. However, gains were smaller in internal validation and not uniformly robust across settings. Incident septic shock analyses were limited by few post-landmark events, particularly in eICU-CRD, and were considered exploratory.

**Conclusion:**

Early longitudinal ABG trajectories in sepsis may capture dynamic physiological heterogeneity and provide limited complementary information beyond conventional clinical variables. Given the modest incremental benefit and inconsistent performance across cohorts, ABG-derived dynamic features should be regarded as adjunctive to clinical severity scores and clinical judgment. Their clinical utility requires prospective validation.

## Introduction

Sepsis is not merely a severe infection; rather, it is a life-threatening syndrome of organ dysfunction caused by a dysregulated host response to infection. Its pathobiology encompasses multiple interrelated processes, including hyperinflammation, immunosuppression, endothelial and microcirculatory injury, and coagulation abnormalities. Accordingly, from the earliest stages of disease onset, sepsis is characterized by pronounced temporal dependence and substantial interindividual heterogeneity (1–6). The 2025 update of the Global Burden of Disease study reported that, in 2021, approximately 166 million sepsis cases occurred worldwide, resulting in 21.4 million sepsis-related deaths and accounting for 31.5% of all global deaths. These findings indicate that sepsis remains one of the most burdensome acute and critical illnesses worldwide (7). More importantly, the clinical burden of sepsis extends beyond its high risk of acute-phase mortality; it also lies in its potential to rapidly precipitate circulatory failure, multiple organ dysfunction, and adverse long-term outcomes. Even among patients who survive to hospital discharge, persistent functional impairment and deterioration in long-term prognosis may still occur (2, 4). Therefore, current international guidelines and recent reviews emphasize that the cornerstone of early sepsis management is timely recognition, source control, antimicrobial therapy, and hemodynamic resuscitation within the first few hours, while accurate risk stratification is essential for determining the timing of intervention, treatment intensity, and allocation of healthcare resources (1, 8–10).

Currently, clinical assessment of sepsis relies primarily on scoring systems such as SOFA, APACHE II, and SAPS II, as well as biomarkers including lactate, procalcitonin, and C-reactive protein. Although these tools have important clinical value, most scoring systems are still based on static information obtained at fixed time points or within short time windows, and therefore may not fully capture the rapidly evolving dynamic heterogeneity of sepsis. Likewise, single biomarkers usually reflect only limited pathophysiological dimensions and are susceptible to the influence of underlying diseases, comorbidities, and therapeutic interventions (3, 13–16). Therefore, risk assessment in sepsis should not be limited to static judgments based on whether a given threshold is reached at a single time point, but should instead move toward risk identification and stratification approaches that can be continuously updated and reflect the dynamic evolution of the disease (10–12).

Arterial blood gas analysis, one of the most time-sensitive and repeatedly obtainable bedside data sources in the ICU, is not only the gold standard for assessing oxygenation but also provides simultaneous information on pH, PaCO_2_, HCO3^−^, base excess, and lactate within the same time window. Therefore, it can comprehensively reflect respiratory function, tissue perfusion, and metabolic acid–base status in patients with sepsis (1). We hypothesized that blood gas parameters and their temporal dynamics may provide valuable prognostic information for sepsis-related outcomes, and that the continuous incorporation of newly accumulated longitudinal data during the disease course may further improve model performance (17). Capturing the dynamic trajectories of blood gas parameters during hospitalization and their temporal associations with sepsis progression may provide a relatively underexplored perspective for understanding sepsis heterogeneity and enabling early risk stratification (11, 12). In recent years, data-driven prediction models have been applied to risk stratification in sepsis; however, how to translate serial blood gas measurements into interpretable and externally verifiable dynamic risk features remains to be further explored. In this study, MIMIC-IV was used as the model development cohort, and eICU-CRD was used as an independent external validation cohort. Based on early repeated ABG measurements, we identified longitudinal blood gas trajectories and evaluated their incremental value and external generalizability for dynamic prognostic prediction in sepsis.

## Materials and methods

### Study design and data sources

This retrospective observational analysis was based on publicly available, de-identified critical care databases and used longitudinal, high-resolution electronic health record data from two large databases, MIMIC-IV and eICU-CRD. MIMIC-IV was pre-specified as the model development cohort, whereas eICU-CRD was pre-specified as the independent external validation cohort. All procedures for trajectory model development, including comparison of candidate class numbers, selection of the final number of classes, specification of spline-based time functions, estimation of model parameters, and rules for calculating posterior assignment probabilities, were performed exclusively using MIMIC-IV. After model development, the final trajectory models were locked and directly applied to eICU-CRD without re-estimating any model parameters. For the dynamic prediction models, MIMIC-IV was further divided into training and internal validation sets using outcome-stratified random sampling for model training, hyperparameter tuning, and internal validation. eICU-CRD was not involved in model training, variable selection, hyperparameter tuning, regression coefficient estimation, or model recalibration, and was used solely to evaluate the predictive performance of the locked models in an independent external validation cohort. Through this design, the present study clearly distinguished internal validation in MIMIC-IV from external validation in eICU-CRD, thereby assessing the stability and generalizability of the dynamic prediction models based on longitudinal blood gas trajectories across different ICU settings.

Specifically, this study used MIMIC-IV version 1.0 and the eICU Collaborative Research Database (18–21). The MIMIC-IV database, released by the Laboratory for Computational Physiology at the Massachusetts Institute of Technology, integrates clinical data from emergency department and ICU patients treated at Beth Israel Deaconess Medical Center between 2008 and 2019, including demographic characteristics, laboratory test results, medication records, vital signs, procedural data, and de-identified clinical notes (18, 20). The eICU Collaborative Research Database (eICU-CRD), jointly maintained by Philips Healthcare and the MIT Laboratory for Computational Physiology, contains data from 200,859 ICU admissions involving 139,367 patients across 335 intensive care units in 208 hospitals in the United States between 2014 and 2015, including vital signs, care plans, severity-of-illness scores, diagnoses, and treatments (19, 21). Other clinical concepts were defined according to the official code resources provided by the databases. This study used only publicly available, de-identified data and involved no direct patient intervention. Data sharing for MIMIC-IV was approved by the Institutional Review Board of Beth Israel Deaconess Medical Center, with a waiver of informed consent.

Sepsis patients were first identified separately from the MIMIC and eICU databases. The study population was then stratified into survivor and non-survivor groups according to in-hospital outcome, and baseline clinical characteristics, comorbidities, supportive therapies, and initial laboratory findings were compared between the two groups. In selecting arterial blood gas variables, both data availability and clinical applicability were taken into account. Specifically, priority was given to blood gas–related variables that were frequently measured within the cohort, had relatively low missingness, and were repeatedly obtainable during the early hospitalization period in patients with sepsis, thereby improving the stability of statistical analyses and the reproducibility of the study findings. Variable selection focused primarily on routinely measured laboratory indicators with broad coverage, with the aim of minimizing the impact of sparse data on longitudinal analyses and predictive modeling (22).

### Study population and cohort construction

Because MIMIC-IV and eICU-CRD differ in data structure, variable recording formats, and sepsis identification strategies, case screening and variable extraction were performed independently within each database. Subsequently, consistent basic inclusion and exclusion criteria were applied across both databases, and the primary trajectory analysis cohorts were constructed according to a unified longitudinal arterial blood gas (ABG) data completeness criterion. In the MIMIC-IV cohort, sepsis was identified using an operationalized Sepsis-3 definition: ICU patients were classified as having sepsis if, in the context of suspected infection, the SOFA assessment time was no earlier than the time of suspected infection and the SOFA score increased by at least 2 points from baseline. The time at which this criterion was met was defined as time zero for the MIMIC-IV cohort. In the eICU-CRD cohort, because the database does not allow direct ascertainment of the exact time of sepsis diagnosis or onset, patients with sepsis were identified according to the APACHE admission diagnosis recorded in the admissiondx table. To restrict the cohort to patients with early ICU sepsis, only cases with a final sepsis diagnosis documented within 36 h after ICU admission were included. Time zero for the eICU-CRD cohort was defined as the time of ICU admission. In both cohorts, only adult ICU patients aged 18 years or older were included, and only the first ICU admission was retained as the unit of analysis.

### Longitudinal ABG data completeness criteria and construction of the trajectory analysis cohort

To ensure the stability of longitudinal trajectory modeling and the estimation of posterior probabilities for individual trajectory class membership, pre-defined completeness criteria for longitudinal arterial blood gas (ABG) data were applied in this study. Using the cohort-specific pre-defined time zero as the reference point, ABG measurements obtained within 0–168 h were partitioned into consecutive 12-h time bins. Patients were included in the primary trajectory analysis cohort only if all eight candidate ABG variables had valid observations in at least three time bins. This criterion was intended to ensure the minimum amount of longitudinal information required for trajectory modeling, rather than to require survival through day 7. Accordingly, patients who died within 168 h could still be included if they had satisfied the repeated-measurement requirement before death. Therefore, the findings of this study should be interpreted as conditional risk prediction applicable to patients with sepsis during the early ICU phase who had sufficiently repeated ABG measurements to support trajectory modeling.

### ABG eligibility and risk-set definition in landmark analysis

During the dynamic prediction phase, this study adopted a landmark analysis framework and constructed prediction tasks at days 3, 5, and 7 after ICU admission, corresponding to 72, 120, and 168 h, respectively. Because estimation of trajectory posterior probabilities required each candidate ABG variable to have valid observations in at least three 12-h time bins, and because a maximum of only two complete time bins could be formed within the first day, no day-1 landmark was defined. Day 3 was therefore pre-specified as the earliest prediction time point.

At each landmark time point, *t*_*L*_– 72, 120, or 168 h—ABG data eligibility and outcome-specific risk-set membership were reassessed within the primary longitudinal ABG trajectory analysis cohort. All predictor variables were generated exclusively from data obtained within the time window from 0 to *t*_*L*_. ABG measurements obtained after *t*_*L*_were not used for trajectory class assignment, posterior probability estimation, feature construction, pre-processing, model training, hyperparameter selection, or model evaluation.

Differences in the number of ABG-eligible patients across landmarks reflected the time-dependent nature of the repeated-measurement requirement; that is, some patients did not meet the data completeness criteria at earlier landmarks but became eligible at subsequent landmarks as pre-landmark ABG data accumulated. For prediction of in-hospital mortality, the risk set was restricted to patients who were alive, remained under hospital observation, and had fulfilled the ABG completeness requirement at the landmark. For prediction of incident septic shock, patients who had developed shock on or before the landmark were additionally excluded. Septic shock was operationally defined according to the Sepsis-3 criteria as the requirement for vasoactive agents or vasopressors to maintain a mean arterial pressure of ≥65 mmHg in conjunction with a lactate concentration >2 mmol/L. For each landmark, the number of ABG-eligible patients, early exclusions, final risk-set size, post-landmark events, and non-events/censored patients were reported. Analyses with an insufficient number of events were interpreted as descriptive or exploratory only.

### Baseline variable collection

To characterize clinical differences associated with in-hospital mortality and to inform subsequent selection of blood gas–related variables and predictive model development, we first compared differences in comorbidity burden, disease severity, supportive therapies, and routine laboratory findings between patients with different outcomes. Baseline characteristic analysis served as an important reference for the subsequent development of in-hospital mortality risk prediction models. Clinical data collected during the early admission period included vital signs, severity-of-illness scores, major comorbidities, supportive therapies, and initial laboratory parameters. Vital signs included heart rate, respiratory rate, body temperature, and mean arterial pressure; systolic blood pressure was additionally included in the eICU cohort. Severity-of-illness scores included APS III and SOFA, with SAPS II additionally collected in the MIMIC cohort. Comorbidities included heart failure, peripheral vascular disease, cerebrovascular disease, chronic pulmonary disease, diabetes mellitus, renal disease, malignancy, and severe liver disease. Laboratory variables included hemoglobin, platelet count, white blood cell count, creatinine, and total bilirubin. Because ABG parameters are influenced by oxygen therapy, mechanical ventilation, and circulatory support strategies, treatment-related variables, including mechanical ventilation, FiO_2_, PaO_2_/FiO_2_, vasoactive agent/vasopressor use, and renal replacement therapy, were incorporated into the models to partially control for the confounding effects of treatment intensity and monitoring intensity on the association between ABG trajectories and outcomes.

### Selection of blood gas variables, time binning, and completeness criteria

Arterial blood gas–related laboratory records were extracted from the MIMIC-IV–derived bg table, and only arterial blood samples were retained. Given the requirements of longitudinal trajectory analysis for repeated measurements and sufficient temporal coverage, candidate variables were selected according to their measurement frequency, degree of missingness, and clinical interpretability within the study cohort. A lollipop plot was used to visually compare the measurement frequencies of all candidate variables. Priority was given to variables that could be repeatedly measured during the early ICU period, had relatively low missingness, and reflected key pathophysiological processes in sepsis, including oxygenation status, ventilatory function, acid–base balance, tissue perfusion, and metabolic abnormalities. Variables with low measurement frequency, excessively sparse time series, limited clinical interpretability, or substantial informational overlap with other selected indicators were excluded from subsequent trajectory analyses.

### Data cleaning and quality control

A data-cleaning strategy prioritizing clinical plausibility and supplemented by statistical rules was applied to exclude observations potentially attributable to data-entry errors, unit inconsistencies, or temporal misalignment, while preserving true extreme values in critically ill patients as far as possible. Rule-based cleaning grounded in medical plausibility was first performed, followed by statistical outlier detection. During pre-processing, biologically implausible zero values were removed. A more permissive outlier-detection approach than the conventional Tukey method was used, based on the 20th and 80th percentiles rather than the traditional 25th and 75th percentiles, to avoid excessive removal of extreme values in skewed distributions. Variable-specific outlier-handling strategies were adopted, and a tiered cleaning framework tailored to arterial blood gas data was established. For variables with relatively stable distributions, for which statistical outliers were more likely to reflect measurement or recording errors—such as pH, PaCO_2_, total CO_2_, base excess, sodium, potassium, and ionized calcium—observations identified as statistical outliers were subsequently treated as missing. For variables substantially influenced by oxygen therapy strategies and mechanical ventilation, such as PaO_2_, the modified Tukey rule was not directly applied to the raw PaO_2_ values; instead, only clinically plausible range checks were performed. For derived oxygenation indices, including the PaO_2_/FiO_2_ ratio and the alveolar–arterial oxygen gradient, the modified Tukey rule was further applied for outlier detection after completion of clinically plausible range checks. Observations identified as either outside the plausible clinical range or statistical outliers were set to missing. For heavy-tailed variables that may carry genuine clinical significance in sepsis, such as lactate and glucose, outlier detection was performed on the log1p-transformed scale; however, these observations were retained as outlier flags only, and the original values were not removed.

### Multiclass trajectory modeling

To characterize heterogeneous dynamic patterns in routine laboratory variables during the early hospitalization period, univariable longitudinal trajectory modeling was performed separately for multiple laboratory indicators. For all variables, measurements obtained within 0–168 h after admission were used for modeling and were divided into 12-h time windows. Within each time window, a representative summary statistic was used as the value for that interval. The time variable was defined as bin_start_h/12, and only patients with observations available in at least three time windows were included.

For each laboratory variable, a two-stage latent trajectory modeling strategy was adopted. In the first stage, latent class growth analysis (LCGA) was used to initially explore temporal patterns in the target variable. The LCGA models did not include random effects and assumed that individuals within the same latent class shared a common mean trajectory shape. The time effect was modeled using natural cubic splines, with internal knots placed at the one-third and two-thirds quantiles of the observation-time distribution for each variable and boundary knots set at the minimum and maximum observed times. During the initial modeling stage, models with one to four latent classes were compared. To reduce the influence of local optima, all multiclass LCGA models were estimated using a multiple-start grid-search procedure. In the second stage, growth mixture models (GMMs) were further constructed on the basis of the LCGA results to refine the trajectory structure. The GMMs retained the same fixed effects and class-specific time-function specification as the LCGA models, while additionally incorporating a linear random effect (random = ~ time) to allow within-class variation in baseline levels and temporal trends. The random-effects covariance matrix was specified as diagonal, and parameter estimation was performed using a multiple-start grid-search procedure. Model selection was based on an integrated assessment of convergence status, Bayesian information criterion (BIC), integrated completed likelihood (ICL), and average posterior probability of assignment (APPA). Only successfully converged models were retained as candidate models. For multiclass models, each class was additionally required to have an APPA of at least 0.70 and to include at least 20 patients. When improvements in fit indices across models with different numbers of classes were limited, the final model preferentially selected was the one with fewer classes, a more parsimonious structure, and more readily interpretable trajectory patterns. Based on the final GMM, the posterior probabilities of assignment (PPAs) to each latent class were extracted for each patient, and class membership was determined according to the maximum posterior probability rule. The complete PPA matrix was also exported for subsequent analyses. To visualize the final trajectory patterns, class-specific predicted mean trajectories over time and their corresponding 95% confidence intervals were calculated. When the model did not directly provide interval estimates, parametric sampling based on the estimated coefficients and their variance–covariance matrix was performed to derive confidence intervals for the predicted trajectories.

All trajectory models were developed and selected exclusively in the MIMIC-IV cohort. After the final GMM was determined, the number of classes, time-function specification, and model parameters were locked and directly applied to the eICU-CRD cohort to calculate posterior probabilities of trajectory class assignment. LCGA or GMM models were not refitted in eICU-CRD, nor were the number of classes reselected or trajectory model parameters re-estimated. For each prediction time point, trajectory posterior probabilities were calculated using only ABG observations available at or before that time point.

### Univariable analysis and heatmap visualization of trajectory subgroups

Patients were stratified according to trajectory class, and demographic characteristics, clinical features, and outcome measures were compared across classes. Continuous variables are presented as medians with interquartile ranges (IQRs) and were compared using the Kruskal–Wallis rank-sum test. Categorical variables are presented as counts and percentages and were compared using Fisher's exact test, with *P*-values estimated based on 2,000 repeated simulations. To account for multiple comparisons, false discovery rate correction was applied to obtain *q*-values. All statistical tests were two-sided, and *P* < 0.05 was considered statistically significant. After completion of the univariable analyses assessing associations between trajectory subgroups and baseline variables, *P*-values were further adjusted using the Benjamini–Hochberg method, and the corresponding *q*-values were reported. The *q*-values for all variables were then summarized into a matrix and visualized as a heatmap, in which *q* < 0.05 was indicated by a red border.

### Development, validation, and incremental value assessment of dynamic prediction models

To evaluate the incremental value of ABG trajectory features beyond conventional clinical risk stratification, four categories of models were developed: (1) a clinical reference model; (2) an ABG trajectory–only model; (3) an ABG trajectory model combined with ABG summary statistics; and (4) a model combining clinical reference variables with ABG features. The clinical reference model included variables such as age, sex, ICU type, initial vital signs, SOFA score, APS III/APACHE-related severity scores, lactate, mechanical ventilation, vasoactive agent/vasopressor use, renal replacement therapy, and major comorbidities. ABG trajectory features comprised the posterior probabilities of assignment (PPAs) to trajectory classes for each blood gas variable, whereas ABG summary statistics included the minimum, maximum, mean, and standard deviation of each blood gas indicator within the corresponding landmark time window. All dynamic prediction models were constructed using elastic-net regularized logistic regression. The MIMIC-IV cohort was stratified by outcome status and randomly split into training and internal validation sets at an 80:20 ratio, with this procedure repeated 25 times for model training, hyperparameter tuning, and internal validation. All feature selection, missing-value imputation, variable encoding, standardization, alpha and lambda selection, and model coefficient estimation were performed exclusively within the MIMIC-IV training set. The feature sets, pre-processing parameters, hyperparameters, and model coefficients determined in the training set were then directly applied to the MIMIC-IV internal validation set and the eICU-CRD external validation cohort, without retraining, hyperparameter tuning, or recalibration in eICU-CRD. Model performance was evaluated using ROC-AUC, PR-AUC, Brier score, calibration intercept, calibration slope, and calibration curves; clinical utility was assessed using decision curve analysis. The incremental value of ABG features was evaluated by comparing the clinical reference model with the combined ABG feature model in terms of discrimination, calibration, overall prediction error, and net benefit.

### Comparison of included and excluded patients and sensitivity analysis

To assess the potential selection bias introduced by the longitudinal ABG completeness requirement, we further compared patients who met the final analysis criteria with patients with sepsis who were excluded from the final analysis because they did not meet the longitudinal ABG data completeness criterion. This comparison was performed separately in the MIMIC-IV and eICU-CRD cohorts. Variables compared included demographic characteristics, ICU type, initial vital signs, severity-of-illness scores, major comorbidities, organ therapies, and outcomes including in-hospital mortality. Continuous variables are presented as medians with interquartile ranges, and categorical variables are presented as counts and percentages. Between-group comparisons were performed using the Wilcoxon rank-sum test, chi-square test, or Fisher's exact test, as appropriate. Absolute standardized mean differences were additionally reported to assess the magnitude of between-group differences.

In addition, to examine whether the main findings depended on the strict eight-variable ABG completeness requirement, we performed a sensitivity analysis in the MIMIC-IV cohort using five core ABG variables: PaO_2_, PaCO_2_, pH, base excess, and lactate. The sensitivity analysis used the same 7-day observation window and 12-h time-bin definition as the main analysis, but required only that each of the five ABG-related variables have at least three valid time bins. Trajectory modeling and dynamic prediction analyses were then repeated, and the baseline characteristics, trajectory structures, and in-hospital mortality prediction performance of the sensitivity analysis cohort were compared with those of the main analysis cohort. All statistical analyses were performed using R version 4.3.3.

## Results

### Study cohort construction and the impact of longitudinal abg completeness screening

**Figure 1:**
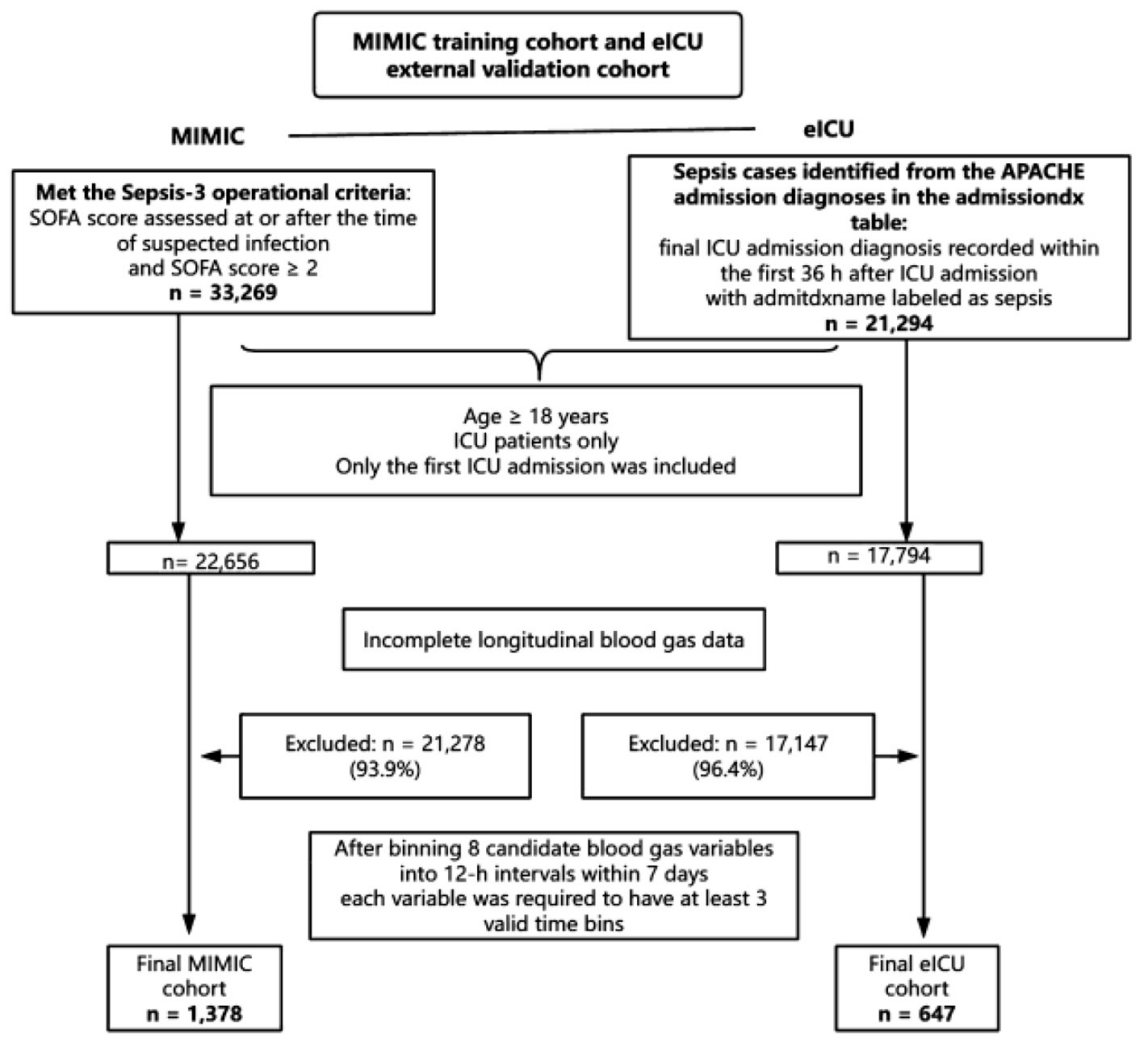
Flowchart of study participant selection. Adult ICU patients with sepsis were identified separately from the MIMIC-IV and eICU-CRD databases. Ultimately, 1,378 patients were included in the MIMIC-IV model development cohort, which was further used for training and internal validation of the dynamic prediction models, and 647 patients were included in the independent eICU-CRD external validation cohort.

Among more than 20 candidate blood gas variables, eight blood gas–related indicators with relatively high data availability and strong clinical interpretability were ultimately selected for subsequent trajectory modeling: PaO_2_, PaCO_2_, FiO_2_, PaO_2_/FiO_2_ ratio, pH, base excess, lactate, and glucose (Supplementary Figure S1). In the MIMIC cohort, 33,269 patients with sepsis were initially identified. After restricting the cohort to patients aged 18 years or older and retaining only the first ICU admission as the unit of analysis, 22,656 patients remained. Following longitudinal blood gas data completeness screening, 1,378 patients were ultimately included in the analysis. In the eICU cohort, 21,294 patients with a diagnosis of sepsis at ICU admission were initially identified. After further restriction to adult patients aged 18 years or older and inclusion of only the first ICU admission, 17,794 patients remained. After application of the same data completeness criteria, 647 patients were ultimately included. The study flowchart is shown in Figure 1.

The longitudinal ABG completeness criterion required each of the eight ABG-related variables to have at least three valid 12-h time windows within the first 7 days after ICU admission. This criterion substantially reduced the final analytic population. Compared with excluded patients, included patients were generally more severely ill, received more organ support, and had a higher risk of in-hospital mortality. In MIMIC-IV, the proportions of assisted ventilation and renal replacement therapy among included patients were 99.5 and 32.4%, respectively, and the in-hospital mortality rate was 26.6%; the corresponding proportions among excluded patients were 53.0, 6.4, and 14.6%, respectively. In eICU-CRD, the proportion of patients with septic shock and the in-hospital mortality rate among included patients were 40.8 and 33.1%, respectively, compared with 20.8 and 16.0% among excluded patients. These findings suggest that the final analytic cohorts were enriched for patients with greater illness severity, longer ICU exposure, and more frequent use of organ support therapies (Supplementary Tables S1, 2).

### Baseline demographic characteristics of the two cohorts

Baseline demographic characteristics are presented in Table 1. In the MIMIC cohort, age, sex, race, and first ICU type differed significantly between in-hospital survivors and non-survivors. Specifically, non-survivors were older than survivors [67 (56, 78) vs. 63 (52, 74) years] and had a lower proportion of males. Differences were also observed in racial composition and distribution of first ICU type; non-survivors were more frequently admitted to the medical ICU, whereas survivors were more commonly admitted to cardiac ICUs. In contrast, in the eICU cohort, only age differed significantly between survivors and non-survivors, with non-survivors similarly being older [67 (58, 75) vs. 61 (50, 70) years]. No significant between-group differences were observed in sex, race, or ICU type distribution.

**Table 1:** Baseline characteristics by database and survival status.

Characteristic	Survivors	Non-survivors	*P*-value	*Q*-value
MIMIC cohort				
Age, years	63 (52, 74)	67 (56, 78)	< 0.001	< 0.001
Gender/sex				
Male	639 (63.1%)	200 (54.6%)	0.003	0.003
Race				
Asian	25 (2.5%)	6 (1.6%)	< 0.001	< 0.001
Black/African American	68 (6.7%)	24 (6.6%)		
Hispanic/Latino	43 (4.2%)	11 (3.0%)		
Other	48 (4.7%)	10 (2.7%)		
White	665 (65.7%)	204 (55.7%)		
Unknown	163 (16.1%)	111 (30.3%)		
First ICU type				
Cardiac ICU	439 (43.4%)	91 (24.9%)	< 0.001	< 0.001
MICU	120 (11.9%)	105 (28.7%)		
SICU	423 (41.8%)	150 (41.0%)		
Other ICU	30 (3.0%)	20 (5.5%)		
eICU cohort				
Age, years	61 (50, 70)	67 (58, 75)	< 0.001	< 0.001
Gender/sex				
Male	223 (50.6%)	102 (47.7%)	0.092	0.126
Female	210 (47.6%)	112 (52.3%)		
Unknown	8 (1.8%)	0 (0.0%)		
Race				
Asian	6 (1.4%)	3 (1.4%)	0.1	0.126
Black/African American	60 (13.6%)	31 (14.5%)		
Hispanic/Latino	12 (2.7%)	3 (1.4%)		
Native American	2 (0.5%)	6 (2.8%)		
Other	18 (4.1%)	12 (5.6%)		
White	331 (75.1%)	157 (73.4%)		
Unknown	12 (2.7%)	2 (0.9%)		
First ICU type				
Cardiac ICU	33 (7.5%)	17 (7.9%)	0.389	0.389
CCU-CTICU	31 (7.0%)	13 (6.1%)		
CSICU	11 (2.5%)	11 (5.1%)		
CTICU	7 (1.6%)	5 (2.3%)		
MICU	81 (18.4%)	36 (16.8%)		
Med-Surg ICU	236 (53.5%)	112 (52.3%)		
Neuro ICU	14 (3.2%)	7 (3.3%)		
SICU	20 (4.5%)	13 (6.1%)		
Unknown	8 (1.8%)	0 (0.0%)		

Layout follows the common Table 1 style used in MIMIC/eICU papers: cohort blocks, subgroup indentation, and p/q columns.

Blank cells indicate that the category was not separately reported in the original uploaded table for that cohort.

Race labels were text-harmonized only (e.g., Caucasian → White; African American → Black/African American); counts were not pooled across categories.

eICU reports Native American separately, whereas Cohort 2 does not provide a separate Native American row.

eICU reports more granular ICU subtypes (CCU-CTICU, CSICU, CTICU, Med-Surg ICU, Neuro ICU); these were retained to preserve the source table structure and original p/q-values.

Length-of-stay metrics differ across the source tables: eICU, ICU length of stay; Cohort 2, hospital length of stay.

### Baseline characteristics and clinical differences associated with in-hospital mortality

Baseline clinical characteristics are presented in Table 2. Overall, non-survivors in the MIMIC cohort had greater illness severity and more pronounced organ dysfunction. Compared with survivors, non-survivors had a higher initial heart rate [93 (79, 109) beats/min vs. 88 (77, 103) beats/min; *q* = 0.008] and a higher respiratory rate [20.0 (17.0, 25.0) breaths/min vs. 18.5 (16.0, 23.0) breaths/min; *q* < 0.001]. In addition, APS III [72 (55, 92) vs. 56 (42, 74); *q* < 0.001], SAPS II [54 (44, 65) vs. 44 (36, 55); *q* < 0.001], and SOFA scores (*q* = 0.008) were all significantly higher among non-survivors, indicating that patients who died were more critically ill during the early ICU admission period. Regarding comorbidities, non-survivors in the MIMIC cohort were more likely to have heart failure, cerebrovascular disease, chronic pulmonary disease, renal disease, and severe liver disease. With respect to supportive therapies, non-survivors had a significantly higher rate of renal replacement therapy, whereas no statistically significant between-group differences were observed in the use of mechanical ventilation or the presence of septic shock. Regarding laboratory findings, non-survivors had higher white blood cell counts, creatinine levels, and total bilirubin levels than survivors, whereas no significant between-group differences were observed in hemoglobin or platelet count. In contrast, fewer differences were observed between survivors and non-survivors in the eICU cohort. After adjustment for multiple comparisons, only initial total bilirubin levels remained significantly higher in non-survivors than in survivors [1.10 (0.60, 2.60) mg/dl vs. 0.80 (0.50, 1.50) mg/dl; *q* = 0.043]. Although APS III, the proportion of patients receiving renal replacement therapy, renal disease, malignancy, and SOFA score showed potential between-group differences in univariable analyses, none remained statistically significant after false discovery rate correction. Overall, clinical characteristics associated with in-hospital mortality were more clearly delineated in the MIMIC cohort, whereas in the eICU cohort, hepatic dysfunction represented the most stable factor associated with adverse outcomes.

**Table 2:** Clinical characteristics by database and survival status.

Characteristic	Survivors	Non-survivors	*P*-value	*Q*-value
MIMIC cohort				
Initial vital signs				
Heart rate, bpm	88 (77, 103)	93 (79, 109)	0.004	0.008
Respiratory rate, breaths/min	18.5 (16.0, 23.0)	20.0 (17.0, 25.0)	< 0.001	< 0.001
Temperature, °C	36.44 (35.70, 37.00)	36.60 (36.22, 36.94)	0.111	0.159
Mean arterial pressure, mmHg	74 (66, 84)	73 (65, 82)	0.288	0.35
Systolic blood pressure, mmHg				
Severity scores				
APS III	56 (42, 74)	72 (55, 92)	< 0.001	< 0.001
SAPS II	44 (36, 55)	54 (44, 65)	< 0.001	< 0.001
SOFA score	4 (3, 6)	4 (3, 6)	0.004	0.008
Comorbidities				
Heart failure	260 (25.7%)	132 (36.1%)	< 0.001	< 0.001
Peripheral vascular disease	241 (23.8%)	79 (21.6%)	0.428	0.492
Cerebrovascular disease	148 (14.6%)	77 (21.0%)	0.006	0.011
Chronic pulmonary disease	252 (24.9%)	118 (32.2%)	0.008	0.014
Diabetes mellitus	310 (30.6%)	119 (32.5%)	0.548	0.601
Renal disease	204 (20.2%)	104 (28.4%)	0.001	0.004
Malignancy	77 (7.6%)	35 (9.6%)	0.289	0.35
Severe liver disease	86 (8.5%)	68 (18.6%)	< 0.001	< 0.001
Supportive therapy/clinical status				
Mechanical ventilation	1,005 (99.3%)	366 (100.0%)	0.2	0.27
Renal replacement therapy	255 (25.2%)	192 (52.5%)	< 0.001	< 0.001
Septic shock	362 (35.8%)	126 (34.4%)	0.691	0.723
Initial laboratory tests				
Hemoglobin, g/dl	10.3 (9.1, 11.7)	10.2 (8.5, 11.7)	0.083	0.128
Platelet count, × 10^9^/L	159.5 (112.8, 230.0)	162.5 (104.2, 242.5)	0.928	0.928
White blood cell count, × 10^9^/L	12.2 (8.2, 17.3)	13.4 (8.7, 20.2)	0.018	0.03
Creatinine, mg/dl	1.1 (0.8, 1.8)	1.6 (1.0, 2.6)	< 0.001	< 0.001
Total bilirubin, mg/dl	0.9 (0.5, 1.8)	1.2 (0.6, 3.0)	< 0.001	< 0.001
eICU cohort				
Initial vital signs				
Heart rate, bpm	103.80 ± 22.33	100.32 ± 22.13	0.078	0.198
Respiratory rate, breaths/min	23.00 (19.00, 28.00)	23.00 (18.00, 27.00)	0.666	0.765
Temperature, °C	37.17 (35.90, 38.10)	37.00 (35.90, 38.20)	0.778	0.852
Mean arterial pressure, mmHg	72.50 (63.25, 81.00)	69.50 (61.00, 80.25)	0.087	0.2
Systolic blood pressure, mmHg	107.00 (88.00, 125.00)	106.00 (86.00, 124.00)	0.629	0.765
Severity scores				
APS III	73.50 (57.25, 96.75)	80.00 (61.00, 102.00)	0.035	0.142
SAPS II				
SOFA score	6.80 ± 3.62	7.49 ± 4.03	0.045	0.146
Comorbidities				
Heart failure	87 (22.5%)	37 (19.5%)	0.409	0.575
Peripheral vascular disease	18 (4.7%)	9 (4.7%)	0.963	0.963
Cerebrovascular disease	33 (8.5%)	26 (13.7%)	0.055	0.157
Chronic pulmonary disease	93 (24.0%)	40 (21.1%)	0.425	0.575
Diabetes mellitus	115 (29.7%)	65 (34.2%)	0.273	0.479
Renal disease	78 (20.2%)	53 (27.9%)	0.037	0.142
Malignancy	69 (17.8%)	48 (25.3%)	0.037	0.142
Severe liver disease	31 (8.0%)	21 (11.1%)	0.23	0.479
Supportive therapy/clinical status				
Mechanical ventilation	125 (32.3%)	68 (35.8%)	0.404	0.575
Renal replacement therapy	101 (26.1%)	66 (34.7%)	0.032	0.142
Septic shock	174 (40.2%)	90 (42.1%)	0.649	0.765
Initial laboratory tests				
Hemoglobin, g/dl	10.40 (9.00, 11.90)	9.90 (8.50, 11.40)	0.009	0.103
Platelet count, × 10^9^/L	170.00 (107.00, 239.50)	163.00 (76.00, 253.00)	0.292	0.479
White blood cell count, × 10^9^/L	13.72 (8.33, 21.01)	13.90 (8.47, 21.12)	0.908	0.949
Creatinine, mg/dl	1.54 (0.91, 2.59)	1.65 (1.03, 2.70)	0.277	0.479
Total bilirubin, mg/dl	0.80 (0.50, 1.50)	1.10 (0.60, 2.60)	0.002	0.043

This sheet follows the same manuscript-style layout as the previously merged table: database blocks, within-block survivor/non-survivor columns, and retained source p/q-values.

Blank cells indicate that the variable was not separately reported in the original uploaded table for that cohort.

Variable names were wording-harmonized only (e.g., Heart failure vs. Congestive heart failure; Dialysis vs. Renal replacement therapy). Source values themselves were not recalculated.

Distribution format differs across source tables and was preserved as reported [e.g., mean ± SD or median (IQR)].

eICU contains Systolic blood pressure, mmHg, which was not separately reported in Cohort 2.

Cohort 2 contains SAPS II, which was not separately reported in the eICU uploaded table.

### Landmark risk sets and outcome event distribution

Before formal dynamic prediction analyses, landmark-specific risk sets were constructed within the primary longitudinal ABG trajectory analysis cohort at days 3, 5, and 7 after ICU admission. This was done to define, at each prediction time point, the number of patients meeting ABG data eligibility criteria, early exclusions, the final analyzable sample size, and the number of post-landmark events. The number of ABG-eligible patients was not fixed across landmarks but varied as pre-landmark ABG data accumulated. In MIMIC-IV, the number of ABG-eligible patients increased from 910 at day 3 to 1,239 at day 5 and 1,378 at day 7; in eICU-CRD, the corresponding numbers increased from 407 to 575 and 647. This pattern primarily reflected that some patients had not yet fulfilled the requirement of at least three valid 12-h time bins for each ABG variable at earlier landmarks, but subsequently met this repeated-measurement criterion at later landmarks. The complete patient flow is presented in Supplementary Table S3.

In-hospital mortality showed a relatively stable event rate after each landmark in both databases, with sufficient numbers of post-landmark events to support formal predictive modeling. In MIMIC-IV, the post-landmark in-hospital mortality rates at the day-3, day-5, and day-7 landmarks were 21.8, 20.0, and 19.0%, respectively, corresponding to 189, 227, and 226 post-landmark death events. In eICU-CRD, the corresponding mortality rates were 23.9, 21.7, and 22.1%, with 84, 89, and 83 events, respectively. Overall, among patients who had survived and remained under observation up to the corresponding landmark, the subsequent risk of in-hospital death remained approximately 19%−24%, indicating that this outcome retained sustained clinical relevance and statistical value for modeling. In contrast, incident septic shock exhibited a distinctly different temporal distribution pattern. A large proportion of shock events had already occurred on or before each landmark, resulting in a markedly reduced risk set available for prediction after incident shock was strictly defined. In MIMIC-IV, the rates of incident shock after the day-3, day-5, and day-7 landmarks were 5.6, 4.3, and 4.6%, respectively, corresponding to 20, 19, and 18 events. In eICU-CRD, the corresponding rates were 2.2, 1.3, and 2.3%, with only 4, 3, and 5 events, respectively. Particularly in the external validation cohort, the number of incident shock events after each landmark was very small, making it difficult to support stable evaluation of model performance and reliable external validation. Based on the above risk-set assessment, subsequent primary prediction analyses focused on in-hospital mortality; incident septic shock was considered a candidate secondary outcome, and its related results were interpreted only as descriptive or exploratory analyses.

### Data availability, time binning, and pre-processing of ABG variables

Among the candidate ABG-related variables, eight parameters were ultimately selected for trajectory modeling: PaO_2_, PaCO_2_, FiO_2_, PaO_2_/FiO_2_ ratio, pH, base excess, lactate, and glucose. All measurements obtained within 0–168 h after ICU admission were partitioned into 12-h time bins, and representative summary values were extracted according to the clinical interpretation of each variable: the maximum value within each time bin was used for lactate, PaCO_2_, FiO_2_, and glucose, whereas the minimum value was used for PaO_2_, PaO_2_/FiO_2_ ratio, pH, and base excess. Data cleaning combined clinically plausible range checks with variable-specific outlier rules. For variables such as pH, PaCO_2_, and base excess, outlying observations were set to missing, whereas for heavy-tailed variables such as lactate and glucose, outliers were flagged but the original values were retained. Detailed bin-level summary rules and outlier-handling thresholds are provided in Supplementary Tables S4, 5.

### Construction and pre-processing of longitudinal ABG data

**Figure 2:**
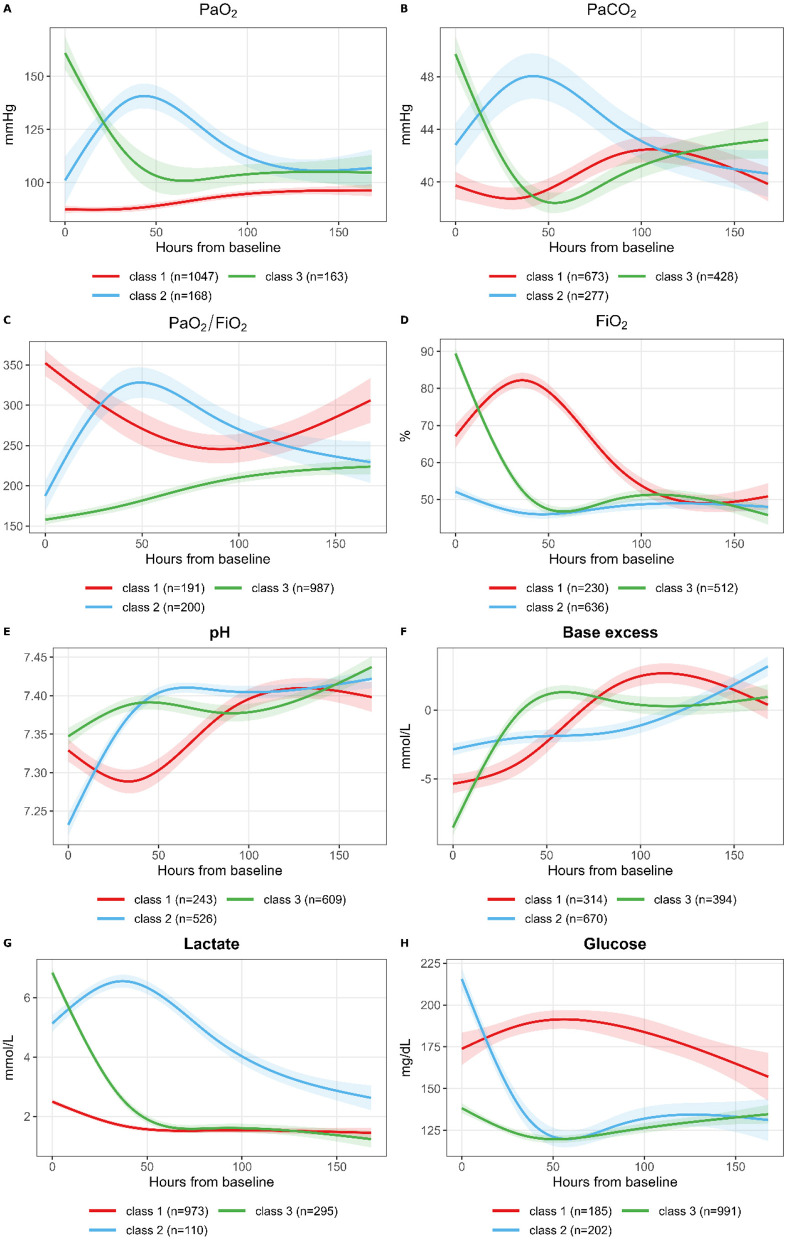
Predicted three-class GMM trajectories for the eight ABG-related variables. **(A–H)** show PaO_2_, PaCO_2_, PaO_2_/FiO_2_ ratio, FiO_2_, pH, base excess, lactate, and glucose, respectively. Curves represent the predicted mean trajectories of each latent trajectory class over 0–168 h after cohort entry, and shaded areas indicate the corresponding 95% confidence intervals. Different colors denote different trajectory classes, with the number of patients in each class shown in parentheses.

Spaghetti plots of the candidate blood gas variables are shown in Supplementary Figure S2. All variables exhibited substantial interindividual heterogeneity and distinct temporal trends during the first 7 days after sepsis onset. Overall, the trajectories of oxygenation- and ventilation-related variables, including FiO_2_, PaO_2_, the PaO_2_/FiO_2_ ratio, and PaCO_2_, were relatively dispersed, whereas pH and base excess showed more concentrated distributions. Lactate exhibited marked early fluctuations and subsequently tended to stabilize, whereas glucose remained highly dispersed throughout the observation period. Taken together, these findings indicate that blood gas variables in patients with sepsis display markedly heterogeneous temporal patterns, providing an intuitive basis for subsequent trajectory modeling. LCGA and GMM trajectory modeling was subsequently performed separately for the eight ABG variables, and candidate models with one to four classes were compared. Model selection was based on a comprehensive assessment of BIC, ICL, average posterior probability of assignment, class size, and clinical interpretability. Although the four-class models showed further improvement in fit indices for some variables, the additional classes generally had small sample sizes or provided limited interpretive gain. Therefore, a more stable and more readily interpretable three-class GMM was ultimately adopted as the primary trajectory model. The complete model search results are presented in Supplementary Table S6. The predicted three-class GMM trajectories for the eight ABG-related variables are shown in Figure 2. Class assignment in the final three-class GMMs was generally acceptable. The minimum APPA across the final models for all variables ranged from 0.745 to 0.948. Specifically, lactate, PaO_2_, PaO_2_/FiO_2_, and glucose each showed one dominant class, with class sizes of 973/110/295, 1,047/168/163, 191/200/987, and 185/202/991, respectively. In contrast, the class distributions for PaCO_2_, FiO_2_, pH, and base excess were relatively more balanced, with class sizes of 673/277/428, 230/636/512, 243/526/609, and 314/670/394, respectively. Overall, early ABG variables in patients with sepsis exhibited distinguishable longitudinal trajectory patterns, and the posterior probabilities of assignment to these trajectory classes were further incorporated as dynamic predictive features in subsequent analyses.

### Univariable associations between trajectory classes and baseline characteristics

**Figure 3:**
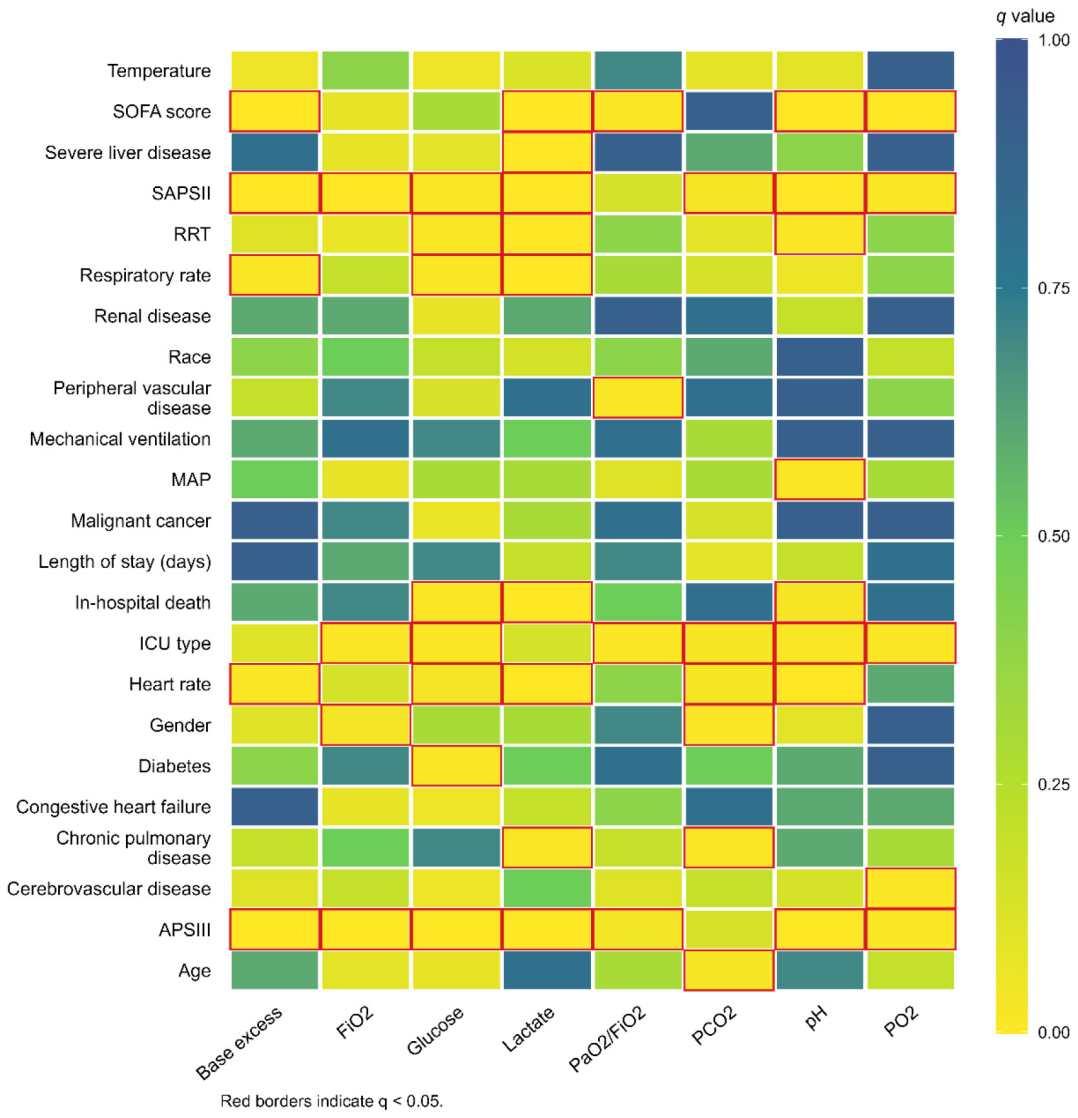
Univariate summary of the associations between different trajectory classes and demographic characteristics, baseline clinical features, and other variables of interest. The results of the univariate analyses are presented as a *q*-value heatmap, in which *q*-values represent *p*-values adjusted for multiple comparisons. Columns correspond to individual blood gas variables, whereas rows represent demographic characteristics, comorbidities, severity scores, vital signs, organ support therapies, and clinical outcomes. Color intensity indicates the magnitude of the *q*-value, and red borders denote *q*-values < 0.05.

To summarize the patterns of association between different blood gas trajectory classes and baseline characteristics, *q*-values after correction for multiple comparisons were visualized as a heatmap (Figure 3), in which red borders indicate *q* < 0.05. Overall, the associations between trajectory classes of different blood gas variables and clinical characteristics were markedly heterogeneous rather than uniform. Indicators of illness severity, particularly APS III, SAPS II, and SOFA scores, were broadly and significantly associated with multiple blood gas variables. Age and length of hospital stay also showed consistent associations with several indicators. In contrast, associations with sex, race, diabetes mellitus, and some hemodynamic variables were more variable and indicator-specific, reaching statistical significance only for selected blood gas trajectories. Organ support therapies and several comorbidities were also associated with specific trajectory classes, suggesting that dynamic blood gas patterns reflect not only the temporal evolution of the biomarkers themselves but also differences in illness severity and clinical phenotypes. Detailed results of the univariable analyses are provided in Supplementary Tables S7–14.

### Dynamic prediction performance for in-hospital mortality: internal validation in MIMIC-IV and external validation in eICU-CRD

**Figure 4:**
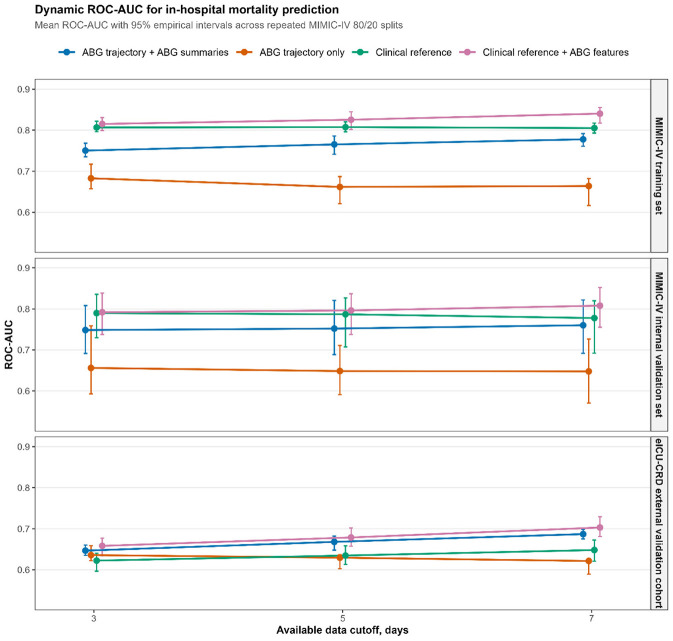
Dynamic ROC-AUC for post-landmark in-hospital mortality prediction. ROC-AUC values of four dynamic prediction models are shown using information available up to the day-3, day-5, and day-7 landmarks in the MIMIC-IV internal validation and eICU-CRD external validation cohorts.

Figure 4 and Table 3 present the performance of in-hospital mortality prediction based on dynamic ABG features. Overall, the combined model incorporating dynamic ABG features demonstrated moderate discriminative ability in both validation settings and showed improvement over the clinical reference model for selected performance metrics. In the MIMIC-IV internal validation set, the model using only posterior probabilities of ABG trajectory membership achieved moderate discrimination. Model performance further improved after the addition of ABG summary statistics, suggesting that trajectory membership itself provided informative risk stratification, while integration of the absolute levels, variability, and phase-specific changes of ABG parameters more comprehensively characterized the risk of in-hospital mortality. After ABG trajectory and summary features were added to the clinical reference model, the ROC-AUCs at days 3, 5, and 7 were 0.792, 0.796, and 0.808, respectively, in the MIMIC-IV internal validation set, and 0.658, 0.679, and 0.703, respectively, in the eICU-CRD external validation cohort. Overall, dynamic ABG features demonstrated moderate predictive ability for in-hospital mortality in both MIMIC-IV internal validation and eICU-CRD external validation, and provided incremental information beyond clinical reference variables. Differences in model performance between the two databases suggest that the relative contributions of trajectory-derived features and numerical summary features may be influenced by differences in patient case-mix, ABG measurement frequency, treatment strategies, and database structure.

**Table 3:** Predictive performance of the clinical reference model and the combined model.

Validation cohort	Landmark day	Model	ROC-AUC	PR-AUC	Brier score	Calibration intercept	Calibration slope
MIMIC-IV internal validation	3	Clinical reference	0.790 (0.730 to 0.836)	0.586 (0.502 to 0.670)	0.162 (0.150 to 0.173)	0.018 (−0.075 to 0.107)	1.869 (0.909 to 3.290)
MIMIC-IV internal validation	3	Clinical reference + ABG features	0.792 (0.738 to 0.839)	0.597 (0.524 to 0.676)	0.157 (0.144 to 0.169)	0.020 (−0.071 to 0.112)	1.541 (1.043 to 2.333)
MIMIC-IV internal validation	5	Clinical reference	0.787 (0.707 to 0.827)	0.580 (0.469 to 0.657)	0.165 (0.152 to 0.177)	0.015 (−0.051 to 0.102)	2.159 (0.969 to 4.190)
MIMIC-IV internal validation	5	Clinical reference + ABG features	0.796 (0.738 to 0.837)	0.608 (0.515 to 0.683)	0.155 (0.141 to 0.168)	0.019 (−0.087 to 0.119)	1.555 (0.964 to 2.219)
MIMIC-IV internal validation	7	Clinical reference	0.778 (0.692 to 0.820)	0.594 (0.454 to 0.663)	0.162 (0.146 to 0.179)	0.015 (−0.092 to 0.096)	1.986 (1.049 to 3.454)
MIMIC-IV internal validation	7	Clinical reference + ABG features	0.808 (0.756 to 0.852)	0.652 (0.558 to 0.722)	0.147 (0.130 to 0.161)	0.020 (−0.095 to 0.113)	1.481 (1.009 to 2.747)
eICU-CRD external validation	3	Clinical reference	0.622 (0.597 to 0.640)	0.473 (0.432 to 0.504)	0.212 (0.208 to 0.216)	0.164 (−0.078 to 0.249)	0.945 (0.709 to 1.148)
eICU-CRD external validation	3	Clinical reference + ABG features	0.658 (0.635 to 0.677)	0.524 (0.486 to 0.541)	0.204 (0.201 to 0.209)	0.147 (−0.007 to 0.280)	1.057 (0.850 to 1.293)
eICU-CRD external validation	5	Clinical reference	0.635 (0.613 to 0.658)	0.492 (0.453 to 0.531)	0.209 (0.202 to 0.213)	0.011 (−0.208 to 0.264)	1.013 (0.678 to 1.247)
eICU-CRD external validation	5	Clinical reference + ABG features	0.679 (0.657 to 0.702)	0.551 (0.511 to 0.576)	0.199 (0.193 to 0.205)	−0.009 (−0.218 to 0.352)	0.912 (0.731 to 1.064)
eICU-CRD external validation	7	Clinical reference	0.648 (0.621 to 0.672)	0.517 (0.464 to 0.545)	0.206 (0.200 to 0.212)	−0.095 (−0.265 to 0.128)	0.857 (0.694 to 1.074)
eICU-CRD external validation	7	Clinical reference + ABG features	0.703 (0.681 to 0.729)	0.583 (0.540 to 0.612)	0.196 (0.186 to 0.205)	−0.141 (−0.423 to 0.062)	0.733 (0.599 to 1.003)

Values are presented as mean (95% empirical interval) across 25 repeated stratified 80:20 splits of the MIMIC-IV cohort.

Models were trained in the 80% MIMIC-IV subset and evaluated in the held-out 20% MIMIC-IV internal validation subset and the independent eICU-CRD external validation cohort.

The combined model denotes the clinical reference model plus arterial blood gas trajectory and summary features.

PR-AUC denotes average precision; ABG, arterial blood gas; eICU-CRD, eICU collaborative research database.

### Incremental value of ABG trajectory features relative to the clinical reference model

To evaluate whether dynamic ABG features provided information beyond standard clinical variables, we compared the clinical reference model with the combined model that incorporated ABG trajectory posterior probabilities and ABG summary statistics. The clinical reference model included age, sex, ICU type, vital signs, SOFA, APS/APACHE-related severity scores, lactate, mechanical ventilation, vasoactive agents/vasopressors, renal replacement therapy, and major comorbidities. In the MIMIC-IV internal validation set, the incremental improvement attributable to ABG features was generally modest. By day 7, the combined model increased the ROC-AUC from 0.778 to 0.808 and the PR-AUC from 0.594 to 0.652, while reducing the Brier score from 0.162 to 0.147; however, the interval estimates for the incremental analyses still crossed zero, indicating uncertainty regarding the observed improvement. In contrast, the incremental value was more consistent in the eICU-CRD external validation cohort. At days 3, 5, and 7, the addition of ABG features increased the ROC-AUC by 0.036, 0.044, and 0.055, respectively, and the PR-AUC by 0.051, 0.059, and 0.066, respectively, while reducing the Brier score by 0.008–0.010. Overall, ABG trajectories and their summary features demonstrated limited incremental predictive value beyond standard clinical risk stratification, and the magnitude of this gain was not fully consistent across validation settings. Therefore, ABG-derived dynamic features may be more appropriately considered as adjunctive information to existing clinical severity scores and clinical judgment (Table 3 and Supplementary Table S15).

### Overall model performance, calibration, and clinical net benefit

**Figure 5:**
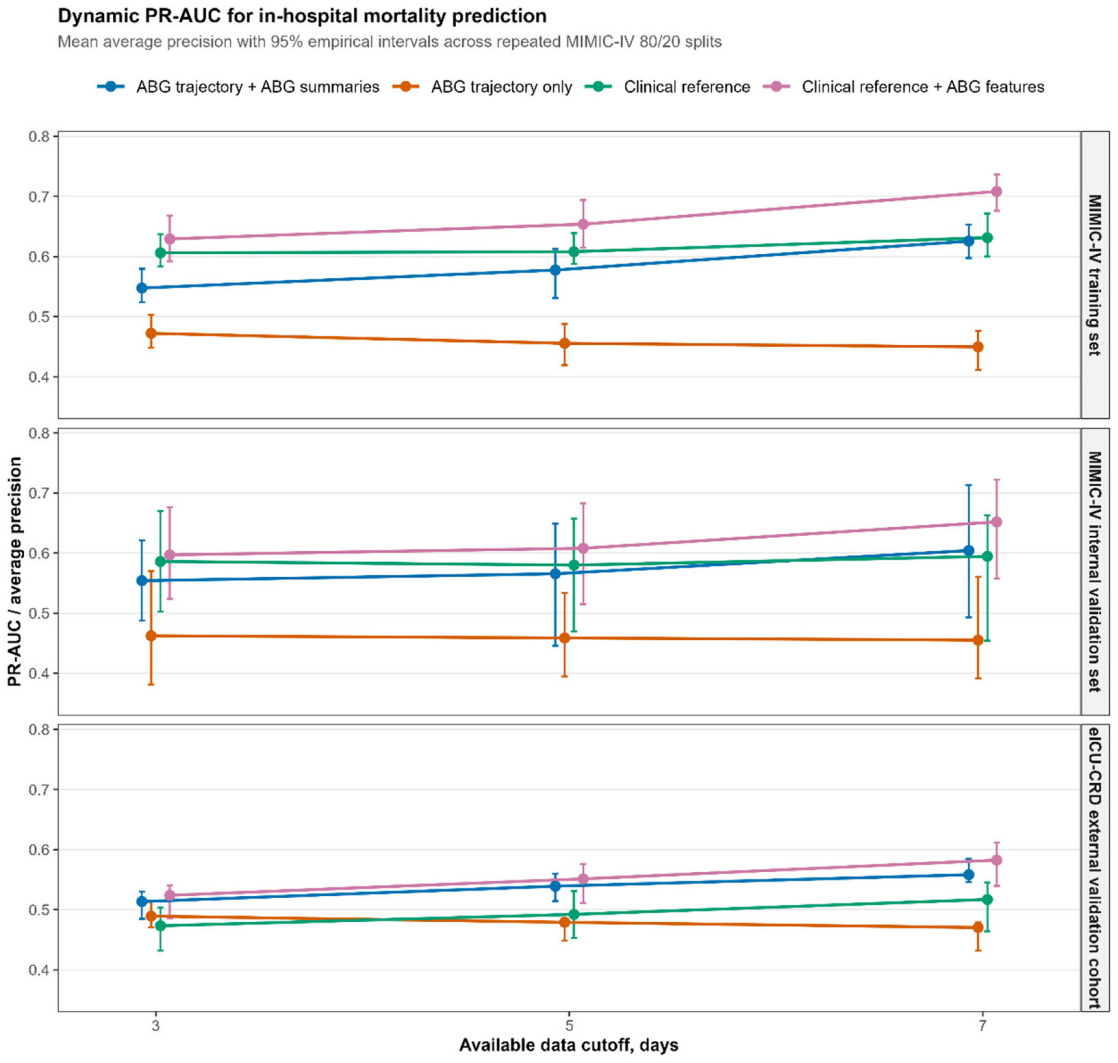
Dynamic PR-AUC for post-landmark in-hospital mortality prediction. PR-AUC values of four dynamic prediction models are shown using information available up to the day-3, day-5, and day-7 landmarks in the MIMIC-IV internal validation and eICU-CRD external validation cohorts.

In addition to ROC-AUC, this study further evaluated PR-AUC, Brier score, calibration performance, and decision curves to comprehensively assess the overall performance and potential clinical utility of the dynamic prediction models. Figure 5 presents the dynamic PR-AUCs of different models for subsequent in-hospital mortality based on information available before the day-3, day-5, and day-7 landmarks. After incorporation of ABG trajectories and summary features, the combined model showed higher PR-AUCs and lower Brier scores across multiple landmarks. In the MIMIC-IV internal validation set, the day-7 combined model increased the PR-AUC from 0.594 to 0.652 and reduced the Brier score from 0.162 to 0.147 compared with the clinical reference model. In the eICU-CRD external validation cohort, the PR-AUCs of the combined model at days 3, 5, and 7 increased from 0.473, 0.492, and 0.517 to 0.524, 0.551, and 0.583, respectively; the corresponding Brier scores decreased from 0.212, 0.209, and 0.206 to 0.204, 0.199, and 0.196, respectively. Calibration analysis showed that the mean predicted risks of the combined model were generally close to the observed risks in the external validation cohort. In eICU-CRD, the calibration intercepts of the combined model at days 3, 5, and 7 were 0.147, −0.009, and −0.141, respectively, and the calibration slopes were 1.057, 0.912, and 0.733, respectively. The calibration slopes at days 3 and 5 were close to 1, whereas the slope decreased at day 7, suggesting a certain degree of calibration deviation in predicted probabilities as the landmark time moved later. Decision curve analysis showed that the combined model provided greater net benefit than the clinical reference model across some clinically relevant threshold ranges, although the overall magnitude of improvement was limited (Supplementary Figures S3, 4).

### Exploratory results for prediction of incident septic shock

In the incident shock analysis, patients who had already developed septic shock on or before each landmark were excluded, and only the risk of new-onset shock after the landmark was evaluated. Unlike in-hospital mortality, incident shock events were concentrated mainly in the early ICU period, resulting in markedly reduced subsequent risk sets and event counts. In MIMIC-IV, the numbers of incident shock events were 20 (5.6%), 19 (4.3%), and 18 (4.6%) at the day-3, day-5, and day-7 landmarks, respectively. In eICU-CRD, only 4 (2.2%), 3 (1.3%), and 5 (2.3%) incident shock events were observed, respectively. In MIMIC-IV, the ROC-AUCs of the model combining ABG trajectories with ABG summary statistics were 0.712, 0.690, and 0.673 at days 3, 5, and 7, respectively; the corresponding ROC-AUCs of the trajectory-only model were 0.670, 0.669, and 0.667. In eICU-CRD, the ROC-AUCs of the combined model were 0.687, 0.689, and 0.688, respectively, whereas those of the trajectory-only model were 0.661, 0.657, and 0.657, respectively (Supplementary Figures S5, 6; Supplementary Tables S16, 17). Given the small number of post-landmark incident shock events, particularly in the eICU-CRD external validation cohort, where only 3–5 events were observed at each landmark, the above ROC-AUC estimates may be subject to substantial statistical uncertainty. Therefore, the prediction results for incident septic shock should be interpreted as descriptive and exploratory only.

### Sensitivity analysis after relaxation of the ABG completeness criterion

To assess whether the main findings depended on the strict eight-variable ABG completeness requirement, we constructed a sensitivity analysis cohort in MIMIC-IV using five core ABG variables: PaO_2_, PaCO_2_, pH, base excess, and lactate. After relaxation of the completeness criterion, the sample size increased from 1,378 patients in the main analysis cohort to 3,772 patients. Compared with the main analysis cohort, the five-variable sensitivity cohort was generally similar in age, sex, APS III, SAPS II, SOFA score, and most comorbidities, but had lower rates of assisted ventilation (92.1 vs. 99.5%) and renal replacement therapy (14.1 vs. 32.4%), suggesting that the relaxed criterion yielded a broader study population with less selective enrichment (Supplementary Table S18). In the five-variable sensitivity analysis, dynamic ABG features retained predictive value for in-hospital mortality. The ROC-AUC of the ABG trajectory plus summary statistics model increased from 0.720 at day 3 to 0.766 at day 7. After ABG features were added to the clinical reference model, the ROC-AUCs at days 3, 5, and 7 were 0.789, 0.799, and 0.824, respectively, all of which were higher than those of the corresponding clinical reference models (0.770, 0.781, and 0.788; Supplementary Figure S7 and Supplementary Table S19). These results were consistent with the direction of the main analysis, suggesting that the principal finding that longitudinal ABG trajectories can be used for in-hospital mortality risk stratification does not entirely depend on simultaneous fulfillment of the completeness requirement for all eight ABG indicators.

## Discussion

Based on two large ICU databases, MIMIC-IV and eICU-CRD, we developed and externally validated a dynamic prognostic prediction framework for sepsis based on ABG trajectories. We found that early ABG parameters in patients with sepsis did not follow a single average pattern of change, but instead exhibited identifiable heterogeneous longitudinal trajectories. As longitudinal information accumulated, the discriminatory ability of dynamic ABG features for in-hospital mortality generally improved. In the formal prediction analyses at days 3, 5, and 7, after ABG trajectories and summary features were added to the clinical reference model, the ROC-AUCs were 0.792, 0.796, and 0.808, respectively, in the MIMIC-IV internal validation set, and 0.658, 0.679, and 0.703, respectively, in the eICU-CRD external validation cohort. Compared with the clinical reference model alone, the addition of ABG features in the eICU-CRD external validation cohort increased the ROC-AUCs at days 3, 5, and 7 by 0.036, 0.044, and 0.055, respectively, indicating that dynamic ABG features can provide additional predictive information for in-hospital mortality risk stratification. In contrast, ABG trajectories showed weaker predictive ability for incident septic shock and did not demonstrate a sustained improvement over time. These findings suggest that ABG trajectories may be better suited to capturing gradually accumulating physiological imbalance and failure of recovery during the course of critical illness, rather than static abnormalities at a single time point. Accordingly, routinely collected ABG data may serve as a dynamic complement to existing risk assessment tools for continuous stratification of in-hospital mortality risk in patients with sepsis (1, 23).

This finding further supports the value of longitudinal physiological information in sepsis risk stratification. Unlike traditional scores or single-time-point biomarkers, which primarily summarize disease severity within a specific time window, ABG trajectories can capture whether abnormalities persist, whether they are corrected, and the direction in which a patient's physiological status evolves. Therefore, the principal methodological significance of this study lies in transforming routinely collected ABG data from static laboratory results into dynamic risk signals that can be updated over time, thereby complementing existing clinical risk assessment frameworks. The association between ABG trajectories and in-hospital mortality may arise from their integrated capture of multisystem physiological dysregulation. PaO_2_, FiO_2_, and PaO_2_/FiO_2_ reflect oxygenation efficiency and the need for oxygen support; PaCO_2_ and pH reflect ventilatory compensation and acid–base homeostasis; and lactate and base excess reflect tissue perfusion, metabolic stress, and buffering reserve. Persistent abnormalities or failure of correction in these indicators may collectively indicate inadequate oxygen delivery, ongoing metabolic derangement, and failure of physiological recovery. The eight ABG-related variables included in this study covered key domains including oxygenation, ventilation, acid–base balance, tissue perfusion, and metabolic stress, enabling ABG trajectories to characterize physiological deterioration during the early course of sepsis more comprehensively than any single blood gas value.

In-hospital mortality is a composite outcome influenced by multiple factors, including oxygenation impairment, ventilatory abnormalities, metabolic acidosis, persistent lactate elevation, intensity of organ support, and the patient's capacity for physiological recovery. Therefore, as longitudinal ABG information accumulates, the model can progressively capture adverse evolution patterns, such as persistent hypoxemia, refractory acid–base imbalance, inadequate lactate clearance, or failure of metabolic compensation. The findings of this study also support this interpretation: as landmark time advanced, the incremental value of ABG features for predicting in-hospital mortality became generally more apparent, whereas prediction of incident shock did not show a similar time-accumulation effect. This may be because shock, as an outcome, is more time-sensitive and treatment-dependent. The diagnosis of shock reflects not only the patient's intrinsic circulatory and metabolic status but also fluid resuscitation, thresholds for vasopressor initiation, monitoring frequency, and clinical documentation practices. In addition, the small number of subsequent incident shock events in the landmark analyses further limited the model's ability to stably learn patterns of shock development. Specifically, the numbers of incident shock events after days 3, 5, and 7 were only 20, 19, and 18, respectively, in MIMIC-IV, and only 4, 3, and 5, respectively, in eICU-CRD. Previous studies have shown that dynamic changes in blood gas–related indicators, such as lactate, pH, and base excess, are closely associated with mortality risk in critically ill patients, and that extreme values or temporal trends within 24–48 h often provide stronger prognostic information than single admission measurements (24). Therefore, our findings further suggest that ABG trajectories may be clinically positioned as dynamic biomarkers of cumulative injury and failure of recovery, rather than merely as early warning indicators for predicting incident shock.

The posterior probabilities of ABG trajectory assignment and ABG summary statistics showed complementary rather than redundant information in predicting in-hospital mortality. Trajectory models can compress serial blood gas measurements into several latent dynamic phenotypes and preserve uncertainty in individual trajectory class membership through posterior probabilities, thereby focusing more on how the disease evolves over time. However, reliance on trajectory classes alone may attenuate the influence of short-term extreme physiological derangements. In patients with sepsis, the lowest pH, highest lactate level, lowest PaO_2_/FiO_2_ ratio, highest FiO_2_ requirement, or marked deviation in base excess often reflects the most severe tissue hypoperfusion, oxygenation failure, or metabolic acid–base disturbance during a given phase of illness (25). Therefore, by jointly modeling trajectory posterior probabilities with the minimum, maximum, mean, and standard deviation of blood gas parameters before the same landmark, this study effectively integrated two dimensions: temporal trends and the magnitude of physiological abnormalities.

Model development, hyperparameter tuning, and internal validation were completed in MIMIC-IV, whereas eICU-CRD was used as a fully independent external validation cohort. eICU-CRD did not participate in variable selection, model training, hyperparameter selection, or recalibration, thereby facilitating assessment of the external generalizability of the ABG trajectory model. Although MIMIC-IV is primarily derived from single-center electronic health records, whereas eICU-CRD includes ICU patients from multiple centers across the United States, and the two databases may differ in case mix, sampling frequency, treatment strategies, and data-recording practices, ABG features still showed relatively consistent incremental improvement over the clinical reference model in eICU-CRD: the ΔROC-AUCs at days 3, 5, and 7 were 0.036, 0.044, and 0.055, respectively; the ΔPR-AUCs were 0.051, 0.059, and 0.066, respectively; and the Brier score decreased at all landmarks. These findings suggest that ABG trajectories may capture dynamic physiological signals that are relatively stable across different ICU settings in patients with sepsis, rather than merely reflecting local patterns within a single database.

At the level of clinical application, the dynamic prediction framework based on longitudinal ABG trajectories is more appropriately positioned as a complementary tool to existing sepsis risk-stratification systems, rather than as a replacement for SOFA, APACHE, or clinical judgment. The value of ABG trajectory modeling lies in transforming routinely collected, repeatable, bedside-accessible blood gas information in the ICU into time-updated risk signals, thereby assisting in the identification of patients with persistent deterioration or inadequate recovery. In this study, ABG trajectories and their summary features provided additional predictive information beyond the clinical reference model, with more consistent performance observed in the eICU-CRD external validation cohort. The combined model improved PR-AUC and reduced the Brier score across multiple landmarks, suggesting that it not only improved risk ranking but may also enhance the accuracy of individualized risk estimation. If its stability is further confirmed through prospective validation and local recalibration, this model could be explored for future integration into electronic health records or ICU information systems as an adjunctive approach for dynamic risk alerting and identification of high-risk patients. Compared with complex black-box machine learning models, the longitudinal trajectory modeling and elastic-net regularized prediction framework used in this study places greater emphasis on interpretability, stability, and cross-cohort generalizability. Trajectory posterior probabilities were used to summarize latent dynamic phenotypes of patients' ABG parameters, whereas the regularized prediction model further integrated clinical variables, trajectory-derived features, and blood gas summary statistics, thereby enabling dynamic risk stratification while avoiding excessive model complexity.

This study has several limitations. First, as a retrospective study based on publicly available critical care databases and electronic health records, it could not fully avoid the effects of documentation bias, missing data, differences in measurement frequency, and unmeasured confounding. The frequency of ABG testing may have been influenced by illness severity, mechanical ventilation, oxygenation targets, vasopressor use, renal replacement therapy, and physician monitoring strategies; therefore, ABG trajectories may simultaneously reflect patients' physiological status, treatment intensity, and monitoring intensity. Although this study incorporated variables such as severity-of-illness scores, lactate, mechanical ventilation, vasoactive agent use, renal replacement therapy, and major comorbidities to adjust for measured confounders, the influence of unmeasured factors, including source control, timeliness of antimicrobial therapy, fluid resuscitation strategies, ventilator settings, and between-center treatment preferences, could not be excluded. Second, this study may be subject to potential bias related to the definition of time zero and the availability of repeated ABG measurements. Because the exact time of sepsis diagnosis or onset could not be directly ascertained in eICU-CRD, ICU admission time was used as the operational time zero for the eICU cohort, which may have resulted in patients being at different stages of disease on day 0. Therefore, the ABG trajectories in this study should be interpreted as dynamic changes in blood gas parameters during the early period after ICU admission among patients with sepsis, rather than as natural disease-course trajectories strictly beginning at the onset or diagnosis of sepsis. In addition, to ensure the stability of trajectory model estimation and individual posterior probability calculation, this study required each candidate ABG variable to cover at least three valid 12-h time bins. This criterion may have enriched the cohort for patients with longer ICU exposure, more frequent monitoring, greater illness severity, or greater use of organ support. Supplementary analyses also showed that included patients had higher disease severity than excluded patients and were markedly enriched for mechanical ventilation and renal replacement therapy. Therefore, the conclusions of this study are primarily applicable to patients with sepsis who have sufficient repeated ABG measurements during the early period after ICU admission, and should not be directly extrapolated to patients with milder illness, less frequent ABG monitoring, or those managed in general wards. Although the landmark framework can reduce the use of future information during predictor construction, it cannot fully eliminate potential bias arising from the definition of time zero, availability of repeated ABG data, ICU exposure time, and differences in monitoring intensity. Third, this study included only eight longitudinal ABG-related variables. Although these variables covered key domains such as oxygenation, ventilation, acid–base balance, tissue perfusion, and metabolic stress, other dynamic information, including inflammation, coagulation, renal function, immune status, and hemodynamics, was not incorporated. Fourth, this study did not systematically compare complex machine learning algorithms, such as random forests, gradient boosting trees, or deep learning models. Elastic-net regularized logistic regression was selected primarily to reduce the risk of overfitting and to improve model interpretability and external transportability. Future studies are needed to compare the performance of different algorithms for modeling dynamic longitudinal ABG features. Fifth, the analysis of incident septic shock should be interpreted with caution. Because most shock events occurred on or before the landmark, the number of post-landmark events was limited after excluding patients with prior shock. Therefore, the related findings should be regarded as descriptive and exploratory only and require validation in independent cohorts with an adequate number of events. Sixth, although external validation in eICU-CRD strengthened the robustness of the findings, both MIMIC-IV and eICU-CRD are derived from ICUs in the United States, and the scope of external validation remains limited. Differences between the two databases in case-mix, sampling frequency, treatment practices, and data-recording patterns may have affected model transportability. Therefore, the findings of this study require further validation across different countries, healthcare systems, hospital levels, and prospective cohorts. Before real-world implementation, local calibration and integration with clinical workflows should also be evaluated. At present, the ABG trajectory model should be regarded as an adjunctive tool for dynamic risk stratification, rather than as a replacement for SOFA, APACHE, or clinical judgment.

## Conclusion

Early arterial blood gas variables in patients with sepsis exhibited marked dynamic heterogeneity, and their longitudinal trajectories captured temporal information that could not be fully reflected by single time-point measurements. For in-hospital mortality prediction, ABG trajectory posterior probabilities and ABG summary statistics demonstrated limited additional predictive value beyond the clinical reference model; however, the magnitude of this incremental improvement was generally small and not fully consistent across validation settings. The analysis of incident septic shock was constrained by the limited number of events and should therefore be interpreted as exploratory only. Accordingly, ABG-derived dynamic features are currently more appropriately considered as adjunctive information to existing clinical severity scores, bedside assessment, and clinical judgment.

## Data Availability

Publicly available datasets were analyzed in this study. This data can be found here: Dataset 1: MIMIC-IV (version 1.0) Direct link: https://physionet.org/content/mimiciv/1.0/ doi: 10.13026/s6n6-xd98 Dataset 2: eICU Collaborative Research Database (eICU-CRD) (version 2.0) Direct link: https://physionet.org/content/eicu-crd/2.0/ doi: 10.13026/C2WM1R.
