# Supplementary material for: Dynamic prognostic prediction in sepsis using longitudinal blood gas trajectories: development and external validation
Source: Front Med (Lausanne). 2026 Jun 12;13:1852841. doi: 10.3389/fmed.2026.1852841 (PMC13303747; doi:10.3389/fmed.2026.1852841)
Supplement: Supplementary file 1 [file Supplementary_file_1.docx]

**Supplementary Materials**

**Supplementary Figure S1.** Non-missing proportion of blood gas biomarkers

**Supplementary Table S1.** Included versus excluded sepsis patients after the longitudinal ABG completeness requirement in the MIMIC-IV cohort

**Supplementary Table S2.** Included versus not included patients after the longitudinal ABG completeness requirement in the eICU-CRD cohort

**Supplementary Table S3.** Landmark-specific eligibility and risk sets
**Supplementary Table S3.1.** Landmark-specific ABG eligibility before risk-set restriction
**Supplementary Table S3.2.** Mortality risk sets and post-landmark deaths by landmark

**Supplementary Table S3.3.** Landmark-specific shock-free risk sets for incident septic shock

**Supplementary Table S4.** Summary statistics used within each 12-hour time bin for blood gas variables

**Supplementary Table S5.** Variable-specific thresholds and outlier handling for arterial blood gas-related variables

**Supplementary Figure S2**. Spaghetti plots of blood gas variables in the modeling dataset during the first 7 days after sepsis onset in the study cohort.

**Supplementary Table S6.** Complete LCGA and GMM model-search results for 1- to 4-class solutions of ABG-related trajectories

**Supplementary Table S7.** Univariate analysis across base excess trajectory classes

**Supplementary Table S8.** Univariate analysis across FiO₂ trajectory classes

**Supplementary Table S9.** Univariate analysis across glucose trajectory classes

**Supplementary Table S10.** Univariate analysis across lactate trajectory classes

**Supplementary Table S11.** Univariate analysis across PaO₂/FiO₂ ratio trajectory classes

**Supplementary Table S12.** Univariate analysis across PaCO₂ trajectory classes

**Supplementary Table S13.** Univariate analysis across pH trajectory classes

**Supplementary Table S14.** Univariate analysis across PaO₂ trajectory classes

**Supplementary Table S15.** Incremental predictive value of ABG trajectory features beyond reference clinical models

**Supplementary Figure S3**. Decision-curve analysis for dynamic prediction of post-landmark in-hospital mortality.

**Supplementary Figure S4. Calibration of dynamic models for post-landmark in-hospital mortality prediction.**

**Supplementary Figure S5.** Exploratory dynamic ROC-AUC for post-landmark incident septic shock prediction in MIMIC-IV

**Supplementary Figure S6.** Exploratory dynamic ROC-AUC for post-landmark incident septic shock prediction in eICU-CRD

**Supplementary Table S16.** Descriptive exploratory ROC-AUC of ABG trajectory models for incident septic shock in MIMIC-IV

**Supplementary Table S17.** Descriptive exploratory ROC-AUC of ABG trajectory models for incident septic shock in eICU-CRD

**Supplementary Table S18.** Baseline characteristics of patients included in the primary cohort and the five-variable sensitivity analysis cohort in MIMIC-IV

**Supplementary Figure S7.** Dynamic ROC-AUC for in-hospital mortality prediction in the five-variable sensitivity cohort

**Supplementary Table S19.** Sensitivity analysis of dynamic prediction for in-hospital mortality using five core ABG variables

Supplementary Figure S1**.Non-missing proportion of blood gas biomarkers**


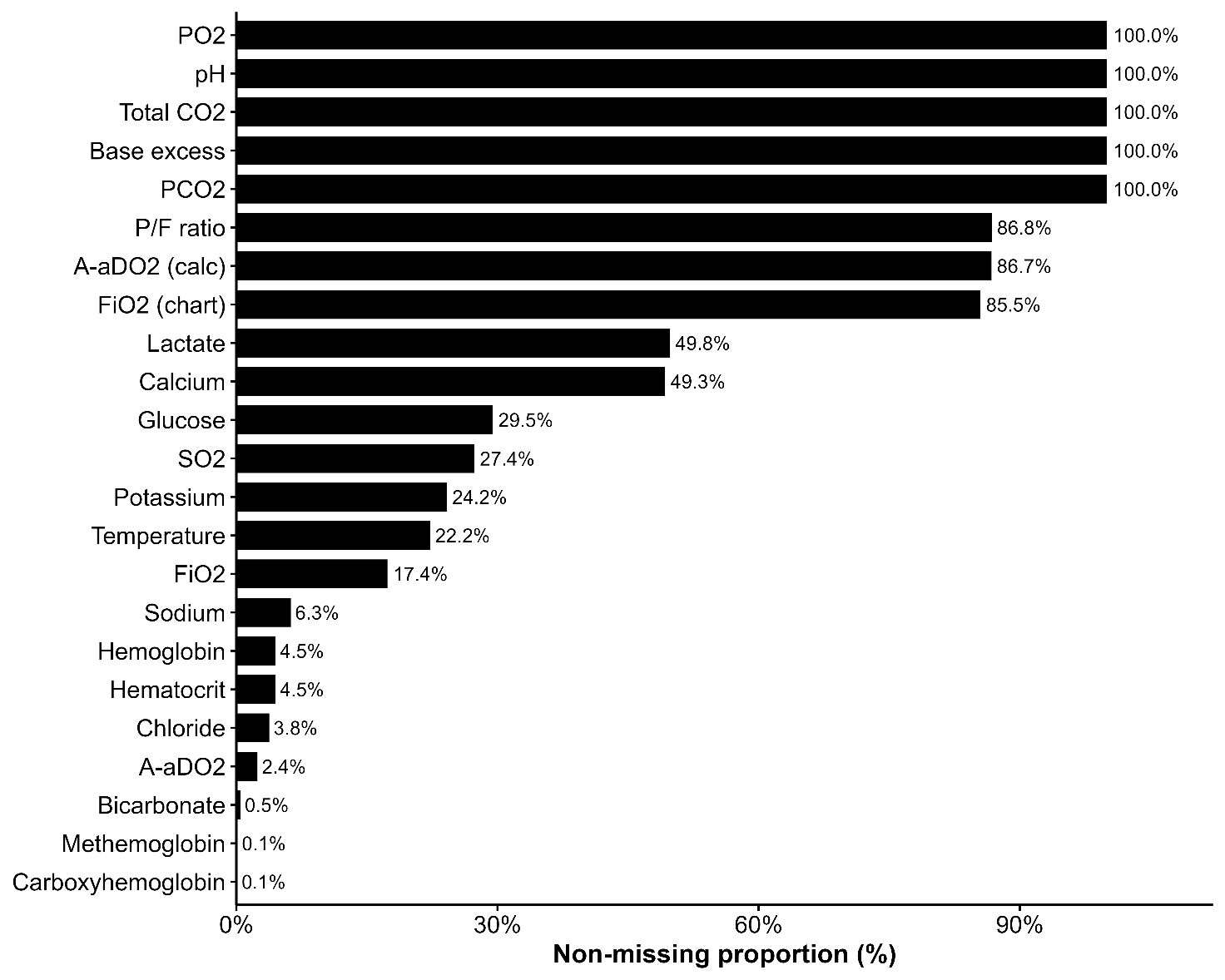


**Supplementary Table S1. Included versus excluded sepsis patients after the longitudinal ABG completeness requirement**

*MIMIC-IV cohort*

| **Characteristic** | **Included cohort** | **Excluded cohort** | **P value** | **SMD** |
| --- | --- | --- | --- | --- |
| No. of patients | 1378 | 21278 |  |  |
| Age, years | 64.00 [52.00, 75.00] | 68.00 [57.00, 79.00] | <0.001 | 0.234 |
| Male sex | 839/1378 (60.9) | 12321/21278 (57.9) | 0.030 | 0.061 |
| **Race** |  |  | <0.001 | 0.148 |
| White | 864/1378 (62.7) | 14186/21278 (66.7) |  |  |
| Black | 92/1378 (6.7) | 1851/21278 (8.7) |  |  |
| Asian | 31/1378 (2.2) | 635/21278 (3.0) |  |  |
| Hispanic/Latino | 54/1378 (3.9) | 695/21278 (3.3) |  |  |
| Other/Unknown | 337/1378 (24.5) | 3911/21278 (18.4) |  |  |
| **ICU type** |  |  | <0.001 | 0.686 |
| Cardiac ICU | 530/1378 (38.5) | 5997/21278 (28.2) |  |  |
| MICU | 225/1378 (16.3) | 9886/21278 (46.5) |  |  |
| SICU | 573/1378 (41.6) | 4778/21278 (22.5) |  |  |
| Other ICU/stepdown/recovery | 50/1378 (3.6) | 617/21278 (2.9) |  |  |
| Hospital length of stay, days | 17.80 [11.03, 27.99] | 7.62 [4.83, 13.00] | <0.001 | 0.766 |
| Heart rate | 89.00 [78.00, 105.00] | 85.00 [74.00, 99.00] | <0.001 | 0.204 |
| Respiratory rate | 19.00 [16.00, 24.00] | 18.00 [16.00, 23.00] | <0.001 | 0.119 |
| Temperature, degrees C | 36.50 [35.80, 37.00] | 36.78 [36.44, 37.17] | <0.001 | 0.406 |
| Mean arterial pressure | 74.00 [66.00, 84.00] | 74.00 [66.00, 85.00] | 0.110 | 0.073 |
| APS III | 60.00 [45.00, 78.00] | 44.00 [33.00, 60.00] | <0.001 | 0.648 |
| SAPS II | 47.00 [37.00, 58.00] | 38.00 [30.00, 47.00] | <0.001 | 0.598 |
| SOFA score | 4.00 [3.00, 6.00] | 3.00 [2.00, 4.00] | <0.001 | 0.534 |
| Congestive heart failure | 392/1378 (28.4) | 5862/21278 (27.5) | 0.470 | 0.020 |
| Peripheral vascular disease | 320/1378 (23.2) | 2297/21278 (10.8) | <0.001 | 0.335 |
| Cerebrovascular disease | 225/1378 (16.3) | 2674/21278 (12.6) | <0.001 | 0.107 |
| Chronic pulmonary disease | 370/1378 (26.9) | 5325/21278 (25.0) | 0.130 | 0.042 |
| Diabetes | 429/1378 (31.1) | 6489/21278 (30.5) | 0.619 | 0.014 |
| Renal disease | 308/1378 (22.4) | 4508/21278 (21.2) | 0.306 | 0.028 |
| Malignant cancer | 112/1378 (8.1) | 3057/21278 (14.4) | <0.001 | 0.198 |
| Severe liver disease | 154/1378 (11.2) | 1542/21278 (7.2) | <0.001 | 0.136 |
| Mechanical ventilation | 1371/1378 (99.5) | 11267/21278 (53.0) | <0.001 | 1.305 |
| Renal replacement therapy | 447/1378 (32.4) | 1371/21278 (6.4) | <0.001 | 0.695 |
| Vasopressor/vasoactive therapy | 508/1378 (36.9) | 10376/21278 (48.8) | <0.001 | 0.242 |
| In-hospital mortality | 366/1378 (26.6) | 3102/21278 (14.6) | <0.001 | 0.300 |

Notes: Continuous variables are summarized as median [IQR]; categorical variables are summarized as n/N (%).

P values are from Wilcoxon rank-sum tests for continuous variables and chi-square tests for categorical variables unless otherwise stated.

SMD = standardized mean difference; ABG = arterial blood gas; SOFA = Sequential Organ Failure Assessment; APS III = Acute Physiology Score III; SAPS II = Simplified Acute Physiology Score II.

Interpretation: the analytical cohort was enriched for higher illness severity, longer exposure, mechanical ventilation, and renal replacement therapy, consistent with selection related to repeated ABG availability.

**Supplementary Table S2. Included versus not included patients after the longitudinal ABG completeness requirement**

*eICU-CRD external validation cohort*

| **Characteristic** | **Included cohort (n = 647)** | **Not included cohort (n = 17,147)** | **P value** | **SMD** |
| --- | --- | --- | --- | --- |
| Age, years | 63.00 [53.00, 72.00] | 68.00 [56.00, 79.00] | <0.001 | -0.305 |
| Heart rate, beats/min | 103.50 [86.00, 118.00] | 97.00 [82.00, 112.00] | <0.001 | 0.226 |
| Respiratory rate, breaths/min | 23.00 [18.00, 28.00] | 22.00 [18.00, 27.00] | 0.074 | 0.047 |
| Temperature, °C | 37.11 [35.90, 38.10] | 37.06 [35.80, 38.10] | 0.802 | -0.150 |
| Mean arterial pressure, mmHg | 71.00 [63.00, 81.00] | 73.00 [62.00, 86.00] | 0.031 | -0.152 |
| SOFA score | 7.00 [4.00, 9.00] | 6.00 [4.00, 8.00] | <0.001 | 0.175 |
| APS III score | 72.00 [56.00, 97.00] | 49.00 [36.00, 66.00] | <0.001 | 0.838 |
| **Sex** |  |  | 0.731 | 0.014 |
| Female | 322/647 (49.8%) | 8413/17141 (49.1%) |  |  |
| Male | 325/647 (50.2%) | 8728/17141 (50.9%) |  |  |
| **Race/ethnicity** |  |  | 0.002 | 0.124 |
| Asian | 9/641 (1.4%) | 349/16964 (2.1%) |  |  |
| Black/African American | 91/641 (14.2%) | 1724/16964 (10.2%) |  |  |
| Hispanic/Latino | 15/641 (2.3%) | 762/16964 (4.5%) |  |  |
| Native American | 8/641 (1.2%) | 146/16964 (0.9%) |  |  |
| Other/Unknown | 30/641 (4.7%) | 838/16964 (4.9%) |  |  |
| White/Caucasian | 488/641 (76.1%) | 13145/16964 (77.5%) |  |  |
| **ICU type** |  |  | <0.001 | 0.278 |
| Cardiac ICU | 50/647 (7.7%) | 1063/17147 (6.2%) |  |  |
| CCU-CTICU | 44/647 (6.8%) | 1032/17147 (6.0%) |  |  |
| CSICU | 22/647 (3.4%) | 264/17147 (1.5%) |  |  |
| CTICU | 12/647 (1.9%) | 127/17147 (0.7%) |  |  |
| Med-Surg ICU | 348/647 (53.8%) | 11531/17147 (67.2%) |  |  |
| MICU | 117/647 (18.1%) | 2196/17147 (12.8%) |  |  |
| Neuro ICU | 21/647 (3.2%) | 326/17147 (1.9%) |  |  |
| SICU | 33/647 (5.1%) | 608/17147 (3.5%) |  |  |
| Septic shock | 264/647 (40.8%) | 3575/17147 (20.8%) | <0.001 | 0.443 |
| In-hospital death | 214/647 (33.1%) | 2745/17147 (16.0%) | <0.001 | 0.405 |
| Heart failure | 124/577 (21.5%) | 3639/17147 (21.2%) | 0.877 | 0.007 |
| Peripheral vascular disease | 27/577 (4.7%) | 917/17147 (5.3%) | 0.482 | -0.031 |
| Cerebrovascular disease | 59/577 (10.2%) | 3407/17147 (19.9%) | <0.001 | -0.272 |
| Chronic pulmonary disease | 133/577 (23.1%) | 4186/17147 (24.4%) | 0.453 | -0.032 |
| Diabetes | 180/577 (31.2%) | 6010/17147 (35.0%) | 0.056 | -0.082 |
| Renal disease | 131/577 (22.7%) | 5993/17147 (35.0%) | <0.001 | -0.273 |
| Malignancy | 117/577 (20.3%) | 3656/17147 (21.3%) | 0.547 | -0.026 |
| Severe liver disease | 52/577 (9.0%) | 875/17147 (5.1%) | <0.001 | 0.153 |
| Mechanical ventilation within 48 h | 193/577 (33.4%) | 5003/17147 (29.2%) | 0.027 | 0.092 |
| Dialysis/RRT within 48 h | 167/577 (28.9%) | 987/17147 (5.8%) | <0.001 | 0.643 |
| Vasoactive agent within 48 h | 96/647 (14.8%) | 4534/17147 (26.4%) | <0.001 | -0.290 |

Notes: Continuous variables are summarized as median [IQR]; categorical variables are summarized as n/N (%).

P values are from Wilcoxon rank-sum tests for continuous variables and chi-square tests for categorical variables.

SMD = standardized mean difference; ABG = arterial blood gas; ICU = intensive care unit; SOFA = Sequential Organ Failure Assessment; APS III = Acute Physiology Score III; RRT = renal replacement therapy.

**Supplementary Table S3** Landmark-specific eligibility and risk sets

Note. Formal trajectory-based landmark prediction was performed at days 3, 5, and 7. At each landmark, ABG eligibility, trajectory posterior probabilities, and ABG summary features were determined exclusively from ABG measurements obtained before or at that landmark. Differences in ABG-eligible sample size across landmarks reflect landmark-specific fulfillment of the repeated-measurement requirement as additional pre-landmark ABG data accumulated. No post-landmark data were used for feature construction, preprocessing, model training, hyperparameter selection, or model evaluation. Final risk sets were further defined by outcome-specific exclusions, and post-landmark events and non-events/censored patients were counted within each corresponding risk set.

Supplementary Table S3.1. Landmark-specific ABG eligibility before risk-set restriction

| Cohort | Landmark | Time, h | Initial cohort, n | ABG-eligible, n (%) |
| --- | --- | --- | --- | --- |
| MIMIC | Day 3 | 72 | 1378 | 910 (66.0) |
| MIMIC | Day 5 | 120 | 1378 | 1239 (89.9) |
| MIMIC | Day 7 | 168 | 1378 | 1378 (100.0) |
| eICU-CRD | Day 3 | 72 | 647 | 407 (62.9) |
| eICU-CRD | Day 5 | 120 | 647 | 575 (88.9) |
| eICU-CRD | Day 7 | 168 | 647 | 647 (100.0) |

Note: ABG eligibility was defined as at least three valid 12-hour bins for each of the eight ABG variables using measurements obtained before or at the corresponding landmark.

Supplementary Table S3.2. Mortality risk sets and post-landmark deaths by landmark

| Cohort | | Landmark | ABG-eligible, n | Pre-landmark exclusions, n (D/C) | Final risk set, n | Death after landmark, n (%) | No death/censored, n |
| --- | --- | --- | --- | --- | --- | --- | --- |
| MIMIC | Day 3 | | 910 | 41/1 | 868 | 189 (21.8) | 679 |
| MIMIC | Day 5 | | 1239 | 93/10 | 1136 | 227 (20.0) | 909 |
| MIMIC | Day 7 | | 1378 | 138/50 | 1190 | 226 (19.0) | 964 |
| eICU-CRD | Day 3 | | 407 | 22/34 | 351 | 84 (23.9) | 267 |
| eICU-CRD | Day 5 | | 575 | 52/113 | 410 | 89 (21.7) | 321 |
| eICU-CRD | Day 7 | | 647 | 73/198 | 376 | 83 (22.1) | 293 |

Note: D denotes deaths before or at the landmark; C denotes discharge/censoring before or at the landmark. The final mortality risk set included ABG-eligible patients who were alive and still under hospital observation at the landmark. Deaths after the landmark and non-events/censored patients were counted within this final risk set.

Supplementary Table S3.3. Landmark-specific shock-free risk sets for incident septic shock

| Cohort | Landmark | ABG-eligible, n | Pre-landmark exclusions, n (S/D/C) | Final shock-free risk set, n | Incident shock after landmark, n (%) | No shock/censored, n |
| --- | --- | --- | --- | --- | --- | --- |
| MIMIC | Day 3 | 910 | 526/12/14 | 358 | 20 (5.6) | 338 |
| MIMIC | Day 5 | 1239 | 689/30/80 | 440 | 19 (4.3) | 421 |
| MIMIC | Day 7 | 1378 | 762/46/181 | 389 | 18 (4.6) | 371 |
| eICU-CRD | Day 3 | 407 | 192/6/28 | 181 | 4 (2.2) | 177 |
| eICU-CRD | Day 5 | 575 | 251/11/82 | 231 | 3 (1.3) | 228 |
| eICU-CRD | Day 7 | 647 | 273/23/137 | 214 | 5 (2.3) | 209 |

Note: S denotes septic shock before or at the landmark; D denotes death before or at the landmark without prior shock; C denotes discharge/censoring before or at the landmark. The final shock-free risk set included ABG-eligible patients who had not developed septic shock and remained under hospital observation at the landmark. Incident shock events after the landmark and non-events/censored patients were counted within this final risk set.

**Supplementary Table S4. Summary statistics used within each 12-hour time bin for blood gas variables**

| **Variable** | **Count variable** | **Bin-level value** | **Summary statistic** | **Clinical rationale** |
| --- | --- | --- | --- | --- |
| Lactate | lactate_n_in_bin | lactate_max | Max | Highest lactate; worst hypoperfusion/metabolic stress |
| PaO₂ | PaO₂_n_in_bin | PaO₂_min | Min | Lowest PaO₂; worst oxygenation level |
| PaCO₂ | PaCO₂_n_in_bin | PaCO₂_max | Max | Highest PaCO₂; worst ventilatory impairment |
| FiO₂ (chart) | fio2_chartevents_n_in_bin | fio2_chartevents_max | Max | Highest FiO₂; maximum oxygen support requirement |
| PaO₂/FiO₂ ratio | PaO₂fio2ratio n_in_bin | PaO₂fio2ratio_min | Min | Lowest PaO₂/FiO₂ ratio; worst oxygenation efficiency |
| pH | ph n_in_bin | ph_min | Min | Lowest pH; most severe acidemia |
| Base excess | baseexcess n_in_bin | baseexcess_min | Min | Lowest base excess; greatest metabolic acid-base derangement |
| Glucose | glucose n_in_bin | glucose_max | Max | Highest glucose; greatest stress hyperglycemia |

Note. Each row of the binned dataset represents one patient-specific 12-hour interval. The n_in_bin variable records the number of measurements available within that interval. The listed bin-level value was used as the representative summary statistic for trajectory modeling.

**Supplementary Table S5. Variable-specific thresholds and outlier handling for arterial blood gas–related variables**

| **Variable (unit)** | **Clinical range** | **Modified Tukey threshold** | **Handling** | **Set missing n (%)** | **Tukey flagged n (%)** |
| --- | --- | --- | --- | --- | --- |
| SO₂ (%) | 1-100 | Not applied | Clinical range only | 0 (0%) | 0 (0%) |
| PaO₂ (mmHg) | 10-700 | Not applied | Clinical range only | 2 (<0.01%) | 0 (0%) |
| PaCO₂ (mmHg) | 5-200 | 11.5-71.5 (raw) | Set missing | 2,652 (1.89%) | 2,648 (1.88%) |
| FiO₂ (chart) (%) | 21-100 | Not applied | Clinical range only | 1 (<0.01%) | 0 (0%) |
| FiO₂ (%) | 21-100 | Not applied | Clinical range only | 0 (0%) | 0 (0%) |
| A-aDO2 (mmHg) | 0-700 | 161-842.6 (raw) | Set missing | 19 (0.51%) | 19 (0.51%) |
| A-aDO2 (calc) (mmHg) | 0-700 | -165.2-572.8 (raw) | Set missing | 4,494 (3.68%) | 2,948 (2.42%) |
| PaO₂/FiO₂ (mmHg) | 20-700 | -122.3-585.4 (raw) | Set missing | 1,519 (1.24%) | 572 (0.47%) |
| pH | 6.5-7.8 | 7.1-7.66 (raw) | Set missing | 1,406 (1.00%) | 1,405 (1.00%) |
| Base excess (mmol/L) | -40-40 | -14.5-13.5 (raw) | Set missing | 4,015 (2.86%) | 4,013 (2.86%) |
| Bicarbonate (mmol/L) | 2-80 | 3.5-39.5 (raw) | Set missing | 4 (0.64%) | 4 (0.64%) |
| Total CO2 (mmol/L) | 2-80 | 7.5-43.5 (raw) | Set missing | 1,682 (1.20%) | 1,681 (1.20%) |
| Hematocrit (%) | 5-75 | Not applied | Clinical range only | 2 (0.03%) | 0 (0%) |
| Hemoglobin (g/dL) | 2-25 | Not applied | Clinical range only | 2 (0.03%) | 0 (0%) |
| CarboxyHb (%) | 0-50 | Not applied | Clinical range only | 0 (0%) | 0 (0%) |
| MetHb (%) | 0-50 | Not applied | Clinical range only | 0 (0%) | 0 (0%) |
| Chloride (mmol/L) | 50-160 | Not applied | Clinical range only | 1 (0.02%) | 0 (0%) |
| Ionized calcium (mmol/L) | 0.2-3.5 | 0.84-1.4 (raw) | Set missing | 1,179 (1.70%) | 1,178 (1.69%) |
| Temperature (C) | 25-45 | Not applied | Clinical range only | 7 (0.02%) | 0 (0%) |
| Potassium (mmol/L) | 1-10 | 2.25-5.85 (raw) | Set missing | 654 (1.94%) | 651 (1.93%) |
| Sodium (mmol/L) | 100-180 | 118.5-154.5 (raw) | Set missing | 233 (2.65%) | 232 (2.64%) |
| Lactate (mmol/L) | 0.1-50 | -0.33-13.1 (log1p; flag only) | Flag only; retained | 1 (<0.01%) | 1,278 (1.83%) |
| Glucose (mg/dL) | 20-1200 | 50.6-333.7 (log1p; flag only) | Flag only; retained | 4 (<0.01%) | 486 (1.18%) |

Note. Percentages were calculated using nonmissing observations before cleaning as the denominator. Clinical range checks were applied before modified Tukey filtering, so the predefined clinical range determined the effective boundary when a statistical Tukey threshold was outside the physiologically plausible range. Modified Tukey thresholds were calculated as P20 - 1.5 × (P80 - P20) and P80 + 1.5 × (P80 - P20). Variables labeled as Set missing were set to missing if they exceeded either the clinical range or the modified Tukey thresholds. Lactate and glucose outliers were detected on the log1p-transformed scale but retained as flag-only values because extreme values may be clinically meaningful in sepsis. Therefore, Tukey-flagged counts are not necessarily equal to observations set missing. Charted FiO₂ was used for trajectory modeling; the additional FiO₂ row is shown for preprocessing transparency. NA, not applicable.


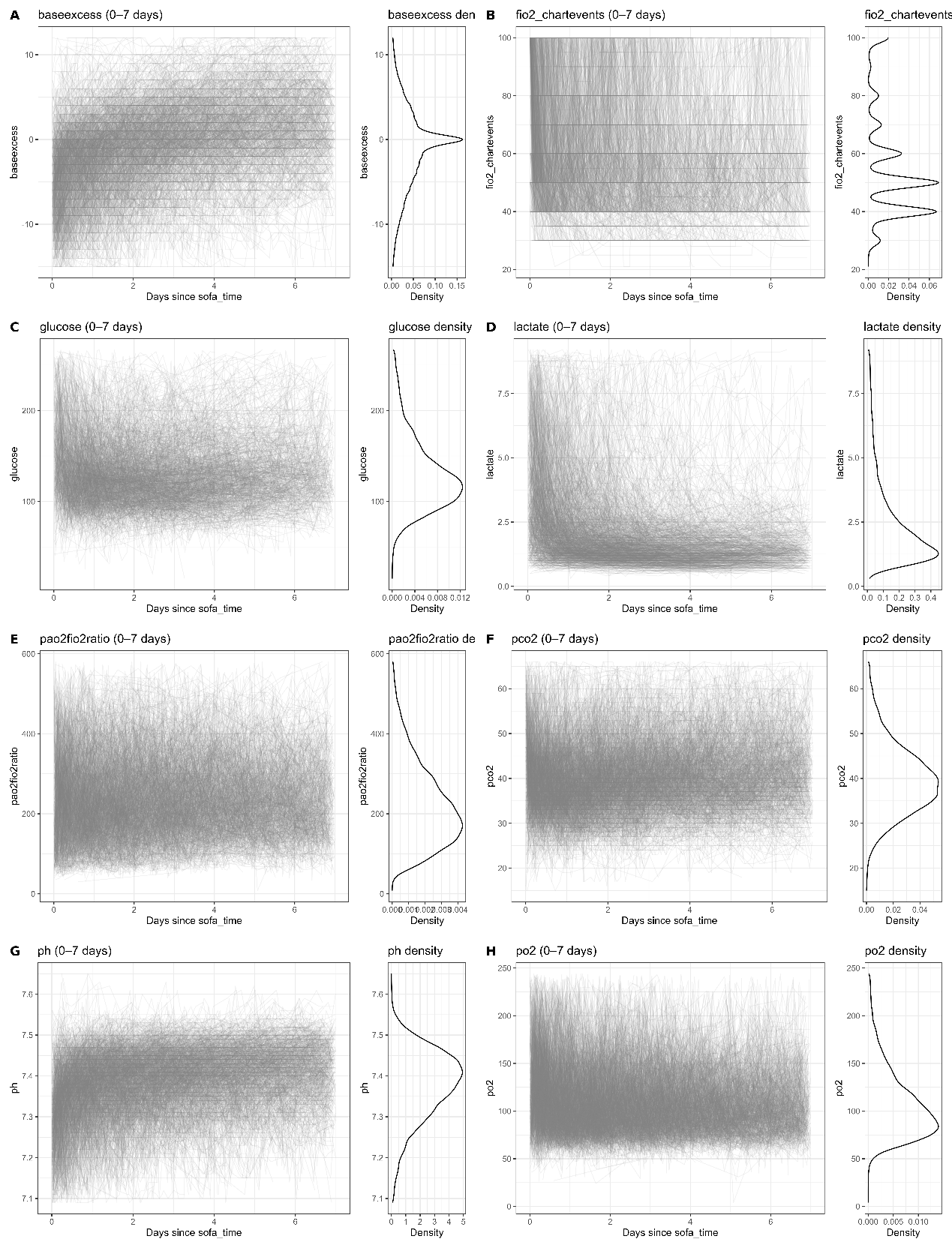


**Supplementary** **Figure S2**. Spaghetti plots of blood gas variables in the modeling dataset during the first 7 days after sepsis onset in the study cohort.

In the main panels, each gray line represents an individual patient and depicts the trajectory of repeated measurements for the corresponding variable during the first 7 days after sepsis onset. The smaller panels on the right show the marginal density distribution of each variable, providing complementary information on its overall distributional characteristics. Together, these plots illustrate the temporal trends of different blood gas parameters during the observation period, as well as the interindividual variability across patients.

**Supplementary Table S6. Complete LCGA and GMM model-search results for 1- to 4-class solutions of ABG-related trajectories**

| **Variable** | **Model** | **Classes** | **AIC** | **BIC** | **ICL** | **Min APPA** | **Class size, n1/n2/n3/n4** |
| --- | --- | --- | --- | --- | --- | --- | --- |
| Lactate | LCGA | 1 | 38150.8 | 38176.9 | 38176.9 | 1.000 | 1378/—/—/— |
| Lactate | LCGA | 2 | 35097.2 | 35149.5 | 35214.2 | 0.958 | 233/1145/—/— |
| **Lactate** | **LCGA** | **3** | **33198.0** | **33276.4** | **33420.2** | **0.925** | **110/919/349/—** |
| Lactate | LCGA | 4 | 32557.6 | 32662.2 | 32813.3 | 0.938 | 39/326/906/107 |
| Lactate | GMM | 1 | 34217.0 | 34253.6 | 34253.6 | 1.000 | 1378/—/—/— |
| Lactate | GMM | 2 | 32774.2 | 32837.0 | 33013.5 | 0.920 | 314/1064/—/— |
| **Lactate** | **GMM** | **3** | **31686.4** | **31775.3** | **31922.8** | **0.948** | **973/110/295/—** |
| Lactate | GMM | 4 | 31276.3 | 31391.3 | 31557.9 | 0.918 | 99/955/60/264 |
| PaO₂ | LCGA | 1 | 131410.2 | 131436.4 | 131436.4 | 1.000 | 1378/—/—/— |
| PaO₂ | LCGA | 2 | 128953.8 | 129006.1 | 129148.1 | 0.917 | 377/1001/—/— |
| **PaO₂** | **LCGA** | **3** | **128436.0** | **128514.5** | **128758.3** | **0.891** | **427/69/882/—** |
| PaO₂ | LCGA | 4 | 127968.5 | 128073.1 | 128416.7 | 0.829 | 849/62/289/178 |
| PaO₂ | GMM | 1 | 128224.9 | 128261.5 | 128261.5 | 1.000 | 1378/—/—/— |
| PaO₂ | GMM | 2 | 127704.0 | 127766.8 | 127950.5 | 0.858 | 1186/192/—/— |
| **PaO₂** | **GMM** | **3** | **127335.9** | **127424.8** | **127724.2** | **0.819** | **1047/168/163/—** |
| PaO₂ | GMM | 4 | 127195.2 | 127310.3 | 127678.3 | 0.789 | 48/978/185/167 |
| PaCO₂ | LCGA | 1 | 95653.4 | 95679.6 | 95679.6 | 1.000 | 1378/—/—/— |
| PaCO₂ | LCGA | 2 | 91944.3 | 91996.6 | 92157.0 | 0.928 | 910/468/—/— |
| **PaCO₂** | **LCGA** | **3** | **90478.0** | **90556.4** | **90791.5** | **0.925** | **146/662/570/—** |
| PaCO₂ | LCGA | 4 | 90055.1 | 90159.6 | 90526.1 | 0.873 | 644/59/408/267 |
| PaCO₂ | GMM | 1 | 89026.5 | 89063.1 | 89063.1 | 1.000 | 1378/—/—/— |
| PaCO₂ | GMM | 2 | 88493.9 | 88556.6 | 89050.6 | 0.821 | 638/740/—/— |
| **PaCO₂** | **GMM** | **3** | **88323.6** | **88412.5** | **89165.0** | **0.745** | **673/277/428/—** |
| PaCO₂ | GMM | 4 | 88223.8 | 88338.8 | 89198.4 | 0.678 | 225/758/228/167 |
| FiO₂ | LCGA | 1 | 110945.8 | 110972.0 | 110972.0 | 1.000 | 1378/—/—/— |
| FiO₂ | LCGA | 2 | 108920.2 | 108972.5 | 109183.3 | 0.908 | 922/456/—/— |
| **FiO₂** | **LCGA** | **3** | **108150.3** | **108228.7** | **108589.1** | **0.865** | **563/534/281/—** |
| FiO₂ | LCGA | 4 | 107667.3 | 107771.8 | 108170.3 | 0.845 | 536/233/488/121 |
| FiO₂ | GMM | 1 | 108295.6 | 108332.2 | 108332.2 | 1.000 | 1378/—/—/— |
| FiO₂ | GMM | 2 | 107624.0 | 107686.7 | 107871.7 | 0.880 | 223/1155/—/— |
| **FiO₂** | **GMM** | **3** | **106915.5** | **107004.3** | **107378.2** | **0.877** | **230/636/512/—** |
| FiO₂ | GMM | 4 | 106718.9 | 106833.9 | 107271.3 | 0.796 | 189/610/130/449 |
| PaO₂/FiO₂ | LCGA | 1 | 152382.4 | 152408.6 | 152408.6 | 1.000 | 1378/—/—/— |
| PaO₂/FiO₂ | LCGA | 2 | 148048.6 | 148100.9 | 148206.7 | 0.948 | 935/443/—/— |
| **PaO₂/FiO₂** | **LCGA** | **3** | **146582.8** | **146661.2** | **146875.7** | **0.926** | **167/675/536/—** |
| PaO₂/FiO₂ | LCGA | 4 | 146088.5 | 146193.1 | 146540.6 | 0.841 | 96/246/619/417 |
| PaO₂/FiO₂ | GMM | 1 | 145371.4 | 145408.0 | 145408.0 | 1.000 | 1378/—/—/— |
| PaO₂/FiO₂ | GMM | 2 | 144948.9 | 145011.6 | 145459.2 | 0.796 | 355/1023/—/— |
| **PaO₂/FiO₂** | **GMM** | **3** | **144553.6** | **144642.5** | **145054.1** | **0.805** | **191/200/987/—** |
| PaO₂/FiO₂ | GMM | 4 | 144331.7 | 144446.7 | 144898.3 | 0.774 | 192/956/168/62 |
| pH | LCGA | 1 | -29698.3 | -29672.1 | -29672.1 | 1.000 | 1378/—/—/— |
| pH | LCGA | 2 | -32989.1 | -32936.8 | -32791.6 | 0.941 | 921/457/—/— |
| **pH** | **LCGA** | **3** | **-33890.2** | **-33811.8** | **-33507.7** | **0.884** | **596/589/193/—** |
| pH | LCGA | 4 | -34443.7 | -34339.2 | -33908.2 | 0.819 | 549/353/306/170 |
| pH | GMM | 1 | -34957.9 | -34921.3 | -34921.3 | 1.000 | 1378/—/—/— |
| pH | GMM | 2 | -35694.2 | -35631.5 | -35194.4 | 0.851 | 689/689/—/— |
| **pH** | **GMM** | **3** | **-35975.8** | **-35886.9** | **-35243.2** | **0.775** | **243/526/609/—** |
| pH | GMM | 4 | -36146.9 | -36031.8 | -35329.5 | 0.785 | 251/265/763/99 |
| Base excess | LCGA | 1 | 79854.6 | 79880.8 | 79880.8 | 1.000 | 1378/—/—/— |
| Base excess | LCGA | 2 | 75357.6 | 75409.9 | 75539.9 | 0.956 | 839/539/—/— |
| **Base excess** | **LCGA** | **3** | **73689.3** | **73767.8** | **73997.9** | **0.914** | **364/690/324/—** |
| Base excess | LCGA | 4 | 72957.8 | 73062.3 | 73436.0 | 0.832 | 270/351/386/371 |
| Base excess | GMM | 1 | 70642.8 | 70679.4 | 70679.4 | 1.000 | 1378/—/—/— |
| Base excess | GMM | 2 | 69549.4 | 69612.2 | 69963.8 | 0.873 | 880/498/—/— |
| **Base excess** | **GMM** | **3** | **69135.4** | **69224.3** | **69832.1** | **0.775** | **314/670/394/—** |
| Base excess | GMM | 4 | 68818.6 | 68933.7 | 69586.9 | 0.776 | 692/105/360/221 |
| Glucose | LCGA | 1 | 83087.9 | 83114.0 | 83114.0 | 1.000 | 1378/—/—/— |
| Glucose | LCGA | 2 | 81507.5 | 81559.8 | 81735.5 | 0.898 | 1074/304/—/— |
| **Glucose** | **LCGA** | **3** | **81161.2** | **81239.7** | **81665.7** | **0.787** | **839/331/208/—** |
| Glucose | LCGA | 4 | 80894.2 | 80998.8 | 81549.0 | 0.773 | 261/118/784/215 |
| Glucose | GMM | 1 | 81185.5 | 81222.1 | 81222.1 | 1.000 | 1378/—/—/— |
| Glucose | GMM | 2 | 80951.5 | 81014.2 | 81454.2 | 0.801 | 222/1156/—/— |
| **Glucose** | **GMM** | **3** | **80711.5** | **80800.4** | **81276.5** | **0.822** | **185/202/991/—** |
| Glucose | GMM | 4 | 80598.5 | 80713.5 | 81291.8 | 0.776 | 167/196/952/63 |

Note. AIC = Akaike information criterion; BIC = Bayesian information criterion; ICL = integrated completed likelihood; APPA = average posterior probability of assignment. Class sizes are shown as n1/n2/n3/n4; em dash indicates not applicable.

**Supplementary Table S7. Univariate analysis across Base excess trajectory classes**

| **Characteristic** | **1** | **2** | **3** | **p-value** | **q-value** |
| --- | --- | --- | --- | --- | --- |
| **Age** | 64.00 (53.00, 75.00) | 63.00 (52.00, 74.00) | 65.00 (52.00, 77.00) | 0.4 | 0.6 |
| **Gender** |  |  |  | 0.050 | 0.11 |
| F | 134 (34%) | 131 (42%) | 274 (41%) |  |  |
| M | 259 (66%) | 183 (58%) | 397 (59%) |  |  |
| **Race** |  |  |  | 0.3 | 0.4 |
| Asian | 12 (3.1%) | 7 (2.2%) | 12 (1.8%) |  |  |
| Black/African American | 24 (6.1%) | 21 (6.7%) | 47 (7.0%) |  |  |
| Hispanic/Latino | 12 (3.1%) | 10 (3.2%) | 32 (4.8%) |  |  |
| Native American | 2 (0.5%) | 1 (0.3%) | 0 (0%) |  |  |
| Other | 38 (9.7%) | 27 (8.6%) | 61 (9.1%) |  |  |
| White | 260 (66%) | 194 (62%) | 410 (61%) |  |  |
| Unknown | 45 (11%) | 54 (17%) | 109 (16%) |  |  |
| **ICU type** |  |  |  | 0.035 | 0.11 |
| Cardiac ICU | 154 (39%) | 108 (34%) | 268 (40%) |  |  |
| General ICU | 0 (0%) | 1 (0.3%) | 0 (0%) |  |  |
| Intermediate/Stepdown | 0 (0%) | 4 (1.3%) | 5 (0.7%) |  |  |
| Med-Surg ICU | 5 (1.3%) | 3 (1.0%) | 7 (1.0%) |  |  |
| MICU | 59 (15%) | 67 (21%) | 99 (15%) |  |  |
| Neuro ICU | 2 (0.5%) | 4 (1.3%) | 14 (2.1%) |  |  |
| PACU/Recovery | 3 (0.8%) | 1 (0.3%) | 1 (0.1%) |  |  |
| SICU | 170 (43%) | 126 (40%) | 277 (41%) |  |  |
| **Length of stay (days)** | 18.06 (11.74, 27.50) | 17.66 (11.15, 28.92) | 17.76 (10.60, 28.31) | 0.9 | >0.9 |
| **Heart rate** | 93.00 (80.00, 108.00) | 91.00 (79.00, 108.00) | 88.00 (76.00, 102.00) | <0.001 | 0.002 |
| Unknown | 12 | 11 | 12 |  |  |
| **Respiratory rate** | 20.00 (16.00, 24.00) | 20.00 (16.00, 24.00) | 18.00 (16.00, 22.00) | <0.001 | 0.003 |
| Unknown | 18 | 13 | 24 |  |  |
| **Temperature** | 36.39 (35.30, 36.78) | 36.61 (36.03, 37.17) | 36.50 (35.80, 37.00) | 0.015 | 0.058 |
| Unknown | 310 | 234 | 485 |  |  |
| **MAP** | 72.00 (65.00, 83.00) | 73.75 (66.00, 83.00) | 74.75 (66.00, 84.00) | 0.3 | 0.5 |
| Unknown | 79 | 62 | 121 |  |  |
| **APSIII** | 69.00 (50.00, 89.00) | 62.00 (47.00, 81.00) | 56.00 (42.00, 72.00) | <0.001 | <0.001 |
| **SAPSII** | 51.00 (40.00, 63.00) | 47.00 (38.00, 58.00) | 45.00 (36.00, 55.00) | <0.001 | <0.001 |
| **SOFA score** | 5.00 (3.00, 7.00) | 4.00 (2.00, 6.00) | 4.00 (2.00, 6.00) | <0.001 | <0.001 |
| **Congestive heart failure** | 111 (28%) | 88 (28%) | 193 (29%) | >0.9 | >0.9 |
| **Peripheral vascular disease** | 98 (25%) | 59 (19%) | 163 (24%) | 0.11 | 0.2 |
| **Cerebrovascular disease** | 49 (12%) | 54 (17%) | 122 (18%) | 0.042 | 0.11 |
| **Chronic pulmonary disease** | 91 (23%) | 83 (26%) | 196 (29%) | 0.11 | 0.2 |
| **Diabetes** | 110 (28%) | 97 (31%) | 222 (33%) | 0.2 | 0.4 |
| **Renal disease** | 96 (24%) | 66 (21%) | 146 (22%) | 0.5 | 0.6 |
| **Malignant cancer** | 35 (8.9%) | 24 (7.6%) | 53 (7.9%) | 0.8 | 0.9 |
| **Severe liver disease** | 48 (12%) | 32 (10%) | 74 (11%) | 0.7 | 0.8 |
| **Mechanical ventilation** | 391 (99%) | 311 (99%) | 669 (100%) | 0.4 | 0.6 |
| **RRT** | 139 (35%) | 112 (36%) | 196 (29%) | 0.046 | 0.11 |
| **In-hospital death** | 99 (25%) | 92 (29%) | 175 (26%) | 0.4 | 0.6 |

Data are presented as median (IQR) or n (%). P values were adjusted for multiple comparisons to obtain q values.

**Supplementary Table S8. Univariate analysis across FiO₂ trajectory classes**

| **Characteristic** | **1** | **2** | **3** | **p-value** | **q-value** |
| --- | --- | --- | --- | --- | --- |
| **Age** | 62.00 (47.00, 73.00) | 65.00 (53.00, 76.00) | 65.00 (53.00, 76.00) | 0.040 | 0.092 |
| **Gender** |  |  |  | 0.005 | 0.032 |
| F | 80 (35%) | 182 (36%) | 277 (44%) |  |  |
| M | 151 (65%) | 330 (64%) | 358 (56%) |  |  |
| **Race** |  |  |  | 0.3 | 0.5 |
| Asian | 5 (2.2%) | 10 (2.0%) | 16 (2.5%) |  |  |
| Black/African American | 18 (7.8%) | 36 (7.0%) | 38 (6.0%) |  |  |
| Hispanic/Latino | 11 (4.8%) | 11 (2.1%) | 32 (5.0%) |  |  |
| Native American | 0 (0%) | 2 (0.4%) | 1 (0.2%) |  |  |
| Other | 15 (6.5%) | 46 (9.0%) | 65 (10%) |  |  |
| White | 143 (62%) | 330 (64%) | 391 (62%) |  |  |
| Unknown | 39 (17%) | 77 (15%) | 92 (14%) |  |  |
| **ICU type** |  |  |  | <0.001 | 0.004 |
| Cardiac ICU | 107 (46%) | 221 (43%) | 202 (32%) |  |  |
| General ICU | 1 (0.4%) | 0 (0%) | 0 (0%) |  |  |
| Intermediate/Stepdown | 0 (0%) | 4 (0.8%) | 5 (0.8%) |  |  |
| Med-Surg ICU | 3 (1.3%) | 5 (1.0%) | 7 (1.1%) |  |  |
| MICU | 40 (17%) | 90 (18%) | 95 (15%) |  |  |
| Neuro ICU | 4 (1.7%) | 1 (0.2%) | 15 (2.4%) |  |  |
| PACU/Recovery | 0 (0%) | 1 (0.2%) | 4 (0.6%) |  |  |
| SICU | 76 (33%) | 190 (37%) | 307 (48%) |  |  |
| **Length of stay (days)** | 18.96 (10.94, 30.83) | 17.50 (10.76, 27.17) | 17.68 (11.11, 28.29) | 0.5 | 0.6 |
| **Heart rate** | 87.00 (73.00, 104.00) | 90.00 (80.00, 106.00) | 90.00 (78.00, 104.00) | 0.068 | 0.14 |
| Unknown | 4 | 15 | 16 |  |  |
| **Respiratory rate** | 19.00 (16.00, 24.00) | 20.00 (16.00, 25.00) | 19.00 (16.00, 23.00) | 0.11 | 0.2 |
| Unknown | 12 | 16 | 27 |  |  |
| **Temperature** | 36.56 (35.90, 37.00) | 36.44 (35.70, 36.83) | 36.50 (35.80, 37.06) | 0.3 | 0.4 |
| Unknown | 154 | 394 | 481 |  |  |
| **MAP** | 75.00 (66.50, 86.00) | 72.00 (65.00, 82.00) | 74.50 (66.00, 84.00) | 0.030 | 0.082 |
| Unknown | 45 | 109 | 108 |  |  |
| **APSIII** | 59.00 (43.00, 75.00) | 65.00 (48.00, 85.00) | 57.00 (43.00, 75.00) | <0.001 | <0.001 |
| **SAPSII** | 43.00 (36.00, 54.00) | 48.00 (40.00, 61.00) | 46.00 (37.00, 56.00) | <0.001 | <0.001 |
| **SOFA score** | 4.00 (3.00, 6.00) | 4.00 (3.00, 6.00) | 4.00 (2.00, 6.00) | 0.025 | 0.082 |
| **Congestive heart failure** | 75 (32%) | 158 (31%) | 159 (25%) | 0.024 | 0.082 |
| **Peripheral vascular disease** | 58 (25%) | 121 (24%) | 141 (22%) | 0.6 | 0.7 |
| **Cerebrovascular disease** | 28 (12%) | 92 (18%) | 105 (17%) | 0.12 | 0.2 |
| **Chronic pulmonary disease** | 71 (31%) | 136 (27%) | 163 (26%) | 0.3 | 0.5 |
| **Diabetes** | 72 (31%) | 166 (32%) | 191 (30%) | 0.7 | 0.7 |
| **Renal disease** | 46 (20%) | 122 (24%) | 140 (22%) | 0.5 | 0.6 |
| **Malignant cancer** | 17 (7.4%) | 38 (7.4%) | 57 (9.0%) | 0.6 | 0.7 |
| **Severe liver disease** | 35 (15%) | 45 (8.8%) | 74 (12%) | 0.032 | 0.082 |
| **Mechanical ventilation** | 231 (100%) | 509 (99%) | 631 (99%) | 0.8 | 0.8 |
| **RRT** | 93 (40%) | 164 (32%) | 190 (30%) | 0.014 | 0.064 |
| **In-hospital death** | 64 (28%) | 140 (27%) | 162 (26%) | 0.7 | 0.7 |

Data are presented as median (IQR) or n (%). P values were adjusted for multiple comparisons to obtain q values.

**Supplementary Table S9. Univariate analysis across Glucose trajectory classes**

| **Characteristic** | **1** | **2** | **3** | **p-value** | **q-value** |
| --- | --- | --- | --- | --- | --- |
| **Age** | 64.00 (51.00, 76.00) | 64.00 (54.00, 71.00) | 66.00 (58.00, 76.00) | 0.047 | 0.083 |
| **Gender** |  |  |  | 0.3 | 0.3 |
| F | 380 (38%) | 82 (44%) | 77 (38%) |  |  |
| M | 610 (62%) | 103 (56%) | 126 (62%) |  |  |
| **Race** |  |  |  | 0.2 | 0.2 |
| Asian | 19 (1.9%) | 6 (3.2%) | 6 (3.0%) |  |  |
| Black/African American | 65 (6.6%) | 12 (6.5%) | 15 (7.4%) |  |  |
| Hispanic/Latino | 35 (3.5%) | 13 (7.0%) | 6 (3.0%) |  |  |
| Native American | 1 (0.1%) | 0 (0%) | 2 (1.0%) |  |  |
| Other | 90 (9.1%) | 17 (9.2%) | 19 (9.4%) |  |  |
| White | 632 (64%) | 103 (56%) | 129 (64%) |  |  |
| Unknown | 148 (15%) | 34 (18%) | 26 (13%) |  |  |
| **ICU type** |  |  |  | <0.001 | 0.002 |
| Cardiac ICU | 397 (40%) | 34 (18%) | 99 (49%) |  |  |
| General ICU | 1 (0.1%) | 0 (0%) | 0 (0%) |  |  |
| Intermediate/Stepdown | 7 (0.7%) | 1 (0.5%) | 1 (0.5%) |  |  |
| Med-Surg ICU | 11 (1.1%) | 4 (2.2%) | 0 (0%) |  |  |
| MICU | 140 (14%) | 63 (34%) | 22 (11%) |  |  |
| Neuro ICU | 12 (1.2%) | 6 (3.2%) | 2 (1.0%) |  |  |
| PACU/Recovery | 4 (0.4%) | 1 (0.5%) | 0 (0%) |  |  |
| SICU | 418 (42%) | 76 (41%) | 79 (39%) |  |  |
| **Length of stay (days)** | 17.84 (11.17, 27.59) | 18.75 (9.44, 31.56) | 16.63 (11.08, 27.24) | 0.6 | 0.7 |
| **Heart rate** | 88.00 (78.00, 105.00) | 90.00 (75.00, 104.00) | 94.00 (80.00, 109.00) | 0.011 | 0.031 |
| Unknown | 27 | 3 | 5 |  |  |
| **Respiratory rate** | 19.00 (16.00, 23.00) | 21.00 (17.00, 25.00) | 19.25 (16.00, 24.00) | <0.001 | 0.002 |
| Unknown | 40 | 8 | 7 |  |  |
| **Temperature** | 36.40 (35.70, 36.94) | 36.78 (36.40, 37.09) | 36.56 (36.00, 37.00) | 0.026 | 0.059 |
| Unknown | 736 | 141 | 152 |  |  |
| **MAP** | 74.50 (66.00, 84.00) | 72.00 (65.00, 83.00) | 73.00 (65.00, 82.00) | 0.3 | 0.3 |
| Unknown | 163 | 59 | 40 |  |  |
| **APSIII** | 57.50 (43.00, 77.00) | 65.00 (53.00, 84.00) | 64.00 (51.00, 78.00) | <0.001 | <0.001 |
| **SAPSII** | 46.00 (37.00, 57.00) | 48.00 (37.00, 59.00) | 50.00 (42.00, 60.00) | <0.001 | 0.002 |
| **SOFA score** | 4.00 (3.00, 6.00) | 4.00 (3.00, 6.00) | 4.00 (3.00, 6.00) | 0.2 | 0.3 |
| **Congestive heart failure** | 261 (26%) | 62 (34%) | 69 (34%) | 0.025 | 0.059 |
| **Peripheral vascular disease** | 232 (23%) | 33 (18%) | 55 (27%) | 0.094 | 0.14 |
| **Cerebrovascular disease** | 154 (16%) | 43 (23%) | 28 (14%) | 0.028 | 0.059 |
| **Chronic pulmonary disease** | 262 (26%) | 48 (26%) | 60 (30%) | 0.7 | 0.7 |
| **Diabetes** | 226 (23%) | 113 (61%) | 90 (44%) | <0.001 | 0.002 |
| **Renal disease** | 204 (21%) | 49 (26%) | 55 (27%) | 0.050 | 0.083 |
| **Malignant cancer** | 72 (7.3%) | 14 (7.6%) | 26 (13%) | 0.036 | 0.070 |
| **Severe liver disease** | 106 (11%) | 30 (16%) | 18 (8.9%) | 0.065 | 0.10 |
| **Mechanical ventilation** | 984 (99%) | 184 (99%) | 203 (100%) | 0.7 | 0.7 |
| **RRT** | 293 (30%) | 91 (49%) | 63 (31%) | <0.001 | 0.002 |
| **In-hospital death** | 240 (24%) | 74 (40%) | 52 (26%) | <0.001 | 0.002 |

Data are presented as median (IQR) or n (%). P values were adjusted for multiple comparisons to obtain q values.

**Supplementary Table S10. Univariate analysis across Lactate trajectory classes**

| **Characteristic** | **1** | **2** | **3** | **p-value** | **q-value** |
| --- | --- | --- | --- | --- | --- |
| **Age** | 65.00 (54.00, 75.00) | 63.50 (47.00, 75.00) | 64.00 (52.00, 76.00) | 0.8 | 0.8 |
| **Gender** |  |  |  | 0.2 | 0.3 |
| F | 116 (39%) | 51 (46%) | 372 (38%) |  |  |
| M | 179 (61%) | 59 (54%) | 601 (62%) |  |  |
| **Race** |  |  |  | 0.077 | 0.15 |
| Asian | 9 (3.1%) | 5 (4.5%) | 17 (1.7%) |  |  |
| Black/African American | 24 (8.1%) | 8 (7.3%) | 60 (6.2%) |  |  |
| Hispanic/Latino | 13 (4.4%) | 8 (7.3%) | 33 (3.4%) |  |  |
| Native American | 2 (0.7%) | 0 (0%) | 1 (0.1%) |  |  |
| Other | 27 (9.2%) | 11 (10%) | 88 (9.0%) |  |  |
| White | 184 (62%) | 58 (53%) | 622 (64%) |  |  |
| Unknown | 36 (12%) | 20 (18%) | 152 (16%) |  |  |
| **ICU type** |  |  |  | 0.070 | 0.15 |
| Cardiac ICU | 126 (43%) | 33 (30%) | 371 (38%) |  |  |
| General ICU | 0 (0%) | 1 (0.9%) | 0 (0%) |  |  |
| Intermediate/Stepdown | 0 (0%) | 0 (0%) | 9 (0.9%) |  |  |
| Med-Surg ICU | 2 (0.7%) | 2 (1.8%) | 11 (1.1%) |  |  |
| MICU | 52 (18%) | 22 (20%) | 151 (16%) |  |  |
| Neuro ICU | 3 (1.0%) | 0 (0%) | 17 (1.7%) |  |  |
| PACU/Recovery | 1 (0.3%) | 1 (0.9%) | 3 (0.3%) |  |  |
| SICU | 111 (38%) | 51 (46%) | 411 (42%) |  |  |
| **Length of stay (days)** | 19.00 (12.17, 29.57) | 18.42 (6.47, 30.07) | 17.55 (10.90, 27.50) | 0.12 | 0.2 |
| **Heart rate** | 95.00 (80.00, 111.00) | 101.00 (83.00, 117.00) | 88.00 (76.00, 101.00) | <0.001 | <0.001 |
| Unknown | 5 | 4 | 26 |  |  |
| **Respiratory rate** | 20.00 (16.00, 25.00) | 20.00 (18.00, 24.00) | 18.50 (16.00, 23.00) | <0.001 | <0.001 |
| Unknown | 11 | 4 | 40 |  |  |
| **Temperature** | 36.40 (35.20, 36.78) | 36.61 (36.22, 36.94) | 36.50 (35.80, 37.06) | 0.054 | 0.12 |
| Unknown | 220 | 72 | 737 |  |  |
| **MAP** | 72.00 (65.00, 83.00) | 73.00 (64.00, 86.00) | 74.00 (66.00, 84.00) | 0.2 | 0.3 |
| Unknown | 60 | 24 | 178 |  |  |
| **APSIII** | 71.00 (53.00, 89.00) | 78.00 (65.00, 99.00) | 55.00 (42.00, 73.00) | <0.001 | <0.001 |
| **SAPSII** | 52.00 (43.00, 63.00) | 54.50 (46.00, 67.00) | 44.00 (36.00, 55.00) | <0.001 | <0.001 |
| **SOFA score** | 5.00 (3.00, 7.00) | 5.00 (3.00, 8.00) | 4.00 (2.00, 6.00) | <0.001 | <0.001 |
| **Congestive heart failure** | 78 (26%) | 41 (37%) | 273 (28%) | 0.092 | 0.2 |
| **Peripheral vascular disease** | 73 (25%) | 24 (22%) | 223 (23%) | 0.8 | 0.8 |
| **Cerebrovascular disease** | 46 (16%) | 13 (12%) | 166 (17%) | 0.4 | 0.5 |
| **Chronic pulmonary disease** | 58 (20%) | 31 (28%) | 281 (29%) | 0.007 | 0.019 |
| **Diabetes** | 88 (30%) | 29 (26%) | 312 (32%) | 0.4 | 0.5 |
| **Renal disease** | 71 (24%) | 27 (25%) | 210 (22%) | 0.6 | 0.6 |
| **Malignant cancer** | 30 (10%) | 11 (10%) | 71 (7.3%) | 0.2 | 0.3 |
| **Severe liver disease** | 43 (15%) | 29 (26%) | 82 (8.4%) | <0.001 | 0.001 |
| **Mechanical ventilation** | 295 (100%) | 110 (100%) | 966 (99%) | 0.4 | 0.5 |
| **RRT** | 121 (41%) | 88 (80%) | 238 (24%) | <0.001 | 0.001 |
| **In-hospital death** | 79 (27%) | 65 (59%) | 222 (23%) | <0.001 | 0.001 |

Data are presented as median (IQR) or n (%). P values were adjusted for multiple comparisons to obtain q values.

**Supplementary Table S11. Univariate analysis across PaO₂/FiO₂ ratio trajectory classes**

| **Characteristic** | **1** | **2** | **3** | **p-value** | **q-value** |
| --- | --- | --- | --- | --- | --- |
| **Age** | 63.00 (50.00, 74.00) | 63.50 (50.50, 75.00) | 65.00 (53.00, 76.00) | 0.15 | 0.3 |
| **Gender** |  |  |  | 0.5 | 0.7 |
| F | 84 (42%) | 79 (41%) | 376 (38%) |  |  |
| M | 115 (58%) | 113 (59%) | 611 (62%) |  |  |
| **Race** |  |  |  | 0.2 | 0.4 |
| Asian | 4 (2.0%) | 5 (2.6%) | 22 (2.2%) |  |  |
| Black/African American | 17 (8.5%) | 15 (7.8%) | 60 (6.1%) |  |  |
| Hispanic/Latino | 6 (3.0%) | 7 (3.6%) | 41 (4.2%) |  |  |
| Native American | 0 (0%) | 1 (0.5%) | 2 (0.2%) |  |  |
| Other | 23 (12%) | 28 (15%) | 75 (7.6%) |  |  |
| White | 120 (60%) | 109 (57%) | 635 (64%) |  |  |
| Unknown | 29 (15%) | 27 (14%) | 152 (15%) |  |  |
| **ICU type** |  |  |  | <0.001 | 0.006 |
| Cardiac ICU | 48 (24%) | 50 (26%) | 432 (44%) |  |  |
| General ICU | 0 (0%) | 0 (0%) | 1 (0.1%) |  |  |
| Intermediate/Stepdown | 0 (0%) | 0 (0%) | 9 (0.9%) |  |  |
| Med-Surg ICU | 2 (1.0%) | 1 (0.5%) | 12 (1.2%) |  |  |
| MICU | 40 (20%) | 19 (9.9%) | 166 (17%) |  |  |
| Neuro ICU | 2 (1.0%) | 7 (3.6%) | 11 (1.1%) |  |  |
| PACU/Recovery | 1 (0.5%) | 1 (0.5%) | 3 (0.3%) |  |  |
| SICU | 106 (53%) | 114 (59%) | 353 (36%) |  |  |
| **Length of stay (days)** | 18.54 (10.93, 29.70) | 18.59 (11.36, 28.64) | 17.55 (10.94, 27.74) | 0.5 | 0.7 |
| **Heart rate** | 93.00 (78.00, 109.00) | 90.00 (75.00, 107.00) | 89.00 (79.00, 104.00) | 0.2 | 0.4 |
| Unknown | 6 | 9 | 20 |  |  |
| **Respiratory rate** | 20.00 (16.00, 24.00) | 19.00 (16.00, 23.00) | 19.00 (16.00, 24.00) | 0.14 | 0.3 |
| Unknown | 6 | 6 | 43 |  |  |
| **Temperature** | 36.44 (35.00, 37.00) | 36.56 (35.94, 37.06) | 36.50 (35.80, 37.00) | 0.5 | 0.7 |
| Unknown | 156 | 150 | 723 |  |  |
| **MAP** | 72.00 (65.00, 81.00) | 77.00 (68.00, 85.00) | 73.00 (65.00, 84.00) | 0.029 | 0.11 |
| Unknown | 44 | 26 | 192 |  |  |
| **APSIII** | 64.00 (50.00, 83.00) | 57.00 (42.00, 75.50) | 60.00 (45.00, 78.00) | 0.007 | 0.038 |
| **SAPSII** | 48.00 (38.00, 57.00) | 45.00 (35.00, 56.00) | 47.00 (38.00, 58.00) | 0.045 | 0.15 |
| **SOFA score** | 4.00 (3.00, 6.00) | 3.00 (2.00, 5.00) | 4.00 (3.00, 6.00) | <0.001 | 0.002 |
| **Congestive heart failure** | 60 (30%) | 45 (23%) | 287 (29%) | 0.2 | 0.4 |
| **Peripheral vascular disease** | 27 (14%) | 46 (24%) | 247 (25%) | 0.002 | 0.015 |
| **Cerebrovascular disease** | 46 (23%) | 28 (15%) | 151 (15%) | 0.024 | 0.11 |
| **Chronic pulmonary disease** | 47 (24%) | 41 (21%) | 282 (29%) | 0.072 | 0.2 |
| **Diabetes** | 63 (32%) | 55 (29%) | 311 (32%) | 0.7 | 0.8 |
| **Renal disease** | 43 (22%) | 45 (23%) | 220 (22%) | >0.9 | >0.9 |
| **Malignant cancer** | 17 (8.5%) | 12 (6.3%) | 83 (8.4%) | 0.6 | 0.8 |
| **Severe liver disease** | 22 (11%) | 22 (11%) | 110 (11%) | >0.9 | >0.9 |
| **Mechanical ventilation** | 199 (100%) | 191 (99%) | 981 (99%) | 0.7 | 0.8 |
| **RRT** | 67 (34%) | 51 (27%) | 329 (33%) | 0.2 | 0.4 |
| **In-hospital death** | 61 (31%) | 50 (26%) | 255 (26%) | 0.4 | 0.5 |

Data are presented as median (IQR) or n (%). P values were adjusted for multiple comparisons to obtain q values.

**Supplementary Table S12. Univariate analysis across PaCO₂ trajectory classes**

| **Characteristic** | **1** | **2** | **3** | **p-value** | **q-value** |
| --- | --- | --- | --- | --- | --- |
| **Age** | 64.00 (53.00, 75.00) | 66.00 (53.00, 77.00) | 62.00 (49.00, 73.00) | 0.006 | 0.029 |
| **Gender** |  |  |  | <0.001 | 0.006 |
| F | 138 (32%) | 302 (45%) | 99 (35%) |  |  |
| M | 291 (68%) | 368 (55%) | 180 (65%) |  |  |
| **Race** |  |  |  | 0.4 | 0.6 |
| Asian | 7 (1.6%) | 20 (3.0%) | 4 (1.4%) |  |  |
| Black/African American | 23 (5.4%) | 53 (7.9%) | 16 (5.7%) |  |  |
| Hispanic/Latino | 18 (4.2%) | 24 (3.6%) | 12 (4.3%) |  |  |
| Native American | 2 (0.5%) | 0 (0%) | 1 (0.4%) |  |  |
| Other | 40 (9.3%) | 63 (9.4%) | 23 (8.2%) |  |  |
| White | 267 (62%) | 412 (61%) | 185 (66%) |  |  |
| Unknown | 72 (17%) | 98 (15%) | 38 (14%) |  |  |
| **ICU type** |  |  |  | 0.003 | 0.023 |
| Cardiac ICU | 188 (44%) | 256 (38%) | 86 (31%) |  |  |
| General ICU | 1 (0.2%) | 0 (0%) | 0 (0%) |  |  |
| Intermediate/Stepdown | 3 (0.7%) | 5 (0.7%) | 1 (0.4%) |  |  |
| Med-Surg ICU | 5 (1.2%) | 5 (0.7%) | 5 (1.8%) |  |  |
| MICU | 72 (17%) | 98 (15%) | 55 (20%) |  |  |
| Neuro ICU | 3 (0.7%) | 16 (2.4%) | 1 (0.4%) |  |  |
| PACU/Recovery | 0 (0%) | 3 (0.4%) | 2 (0.7%) |  |  |
| SICU | 157 (37%) | 287 (43%) | 129 (46%) |  |  |
| **Length of stay (days)** | 16.38 (10.28, 27.59) | 17.68 (10.74, 27.58) | 19.69 (12.22, 29.64) | 0.028 | 0.085 |
| **Heart rate** | 90.00 (80.00, 108.00) | 88.00 (75.00, 103.00) | 91.00 (79.00, 107.00) | 0.008 | 0.030 |
| Unknown | 9 | 21 | 5 |  |  |
| **Respiratory rate** | 19.00 (16.00, 24.00) | 19.00 (16.00, 23.00) | 20.00 (16.00, 25.00) | 0.072 | 0.14 |
| Unknown | 15 | 25 | 15 |  |  |
| **Temperature** | 36.44 (35.83, 36.94) | 36.40 (35.70, 37.00) | 36.66 (36.39, 37.06) | 0.030 | 0.085 |
| Unknown | 336 | 480 | 213 |  |  |
| **MAP** | 72.00 (65.00, 84.00) | 74.75 (66.00, 83.00) | 74.50 (66.00, 85.00) | 0.2 | 0.3 |
| Unknown | 92 | 126 | 44 |  |  |
| **APSIII** | 61.00 (46.00, 81.00) | 59.00 (43.00, 78.00) | 60.00 (47.00, 75.00) | 0.069 | 0.14 |
| **SAPSII** | 48.00 (40.00, 59.00) | 47.00 (37.00, 57.00) | 45.00 (36.00, 57.00) | 0.006 | 0.029 |
| **SOFA score** | 4.00 (3.00, 6.00) | 4.00 (3.00, 6.00) | 4.00 (3.00, 6.00) | >0.9 | >0.9 |
| **Congestive heart failure** | 128 (30%) | 185 (28%) | 79 (28%) | 0.7 | 0.8 |
| **Peripheral vascular disease** | 105 (24%) | 154 (23%) | 61 (22%) | 0.7 | 0.8 |
| **Cerebrovascular disease** | 76 (18%) | 115 (17%) | 34 (12%) | 0.090 | 0.2 |
| **Chronic pulmonary disease** | 136 (32%) | 141 (21%) | 93 (33%) | <0.001 | 0.006 |
| **Diabetes** | 123 (29%) | 220 (33%) | 86 (31%) | 0.4 | 0.5 |
| **Renal disease** | 95 (22%) | 145 (22%) | 68 (24%) | 0.6 | 0.8 |
| **Malignant cancer** | 30 (7.0%) | 50 (7.5%) | 32 (11%) | 0.073 | 0.14 |
| **Severe liver disease** | 41 (9.6%) | 81 (12%) | 32 (11%) | 0.4 | 0.6 |
| **Mechanical ventilation** | 429 (100%) | 665 (99%) | 277 (99%) | 0.2 | 0.3 |
| **RRT** | 150 (35%) | 195 (29%) | 102 (37%) | 0.036 | 0.093 |
| **In-hospital death** | 120 (28%) | 174 (26%) | 72 (26%) | 0.7 | 0.8 |

Data are presented as median (IQR) or n (%). P values were adjusted for multiple comparisons to obtain q values.

**Supplementary Table S13. Univariate analysis across pH trajectory classes**

| **Characteristic** | **1** | **2** | **3** | **p-value** | **q-value** |
| --- | --- | --- | --- | --- | --- |
| **Age** | 66.00 (56.00, 75.00) | 64.00 (53.00, 75.00) | 64.00 (52.00, 76.00) | 0.6 | 0.7 |
| **Gender** |  |  |  | 0.042 | 0.093 |
| F | 102 (42%) | 185 (35%) | 252 (42%) |  |  |
| M | 141 (58%) | 345 (65%) | 353 (58%) |  |  |
| **Race** |  |  |  | 0.8 | 0.9 |
| Asian | 7 (2.9%) | 13 (2.5%) | 11 (1.8%) |  |  |
| Black/African American | 19 (7.8%) | 34 (6.4%) | 39 (6.4%) |  |  |
| Hispanic/Latino | 10 (4.1%) | 18 (3.4%) | 26 (4.3%) |  |  |
| Native American | 0 (0%) | 3 (0.6%) | 0 (0%) |  |  |
| Other | 21 (8.6%) | 42 (7.9%) | 63 (10%) |  |  |
| White | 147 (60%) | 339 (64%) | 378 (62%) |  |  |
| Unknown | 39 (16%) | 81 (15%) | 88 (15%) |  |  |
| **ICU type** |  |  |  | 0.004 | 0.015 |
| Cardiac ICU | 83 (34%) | 205 (39%) | 242 (40%) |  |  |
| General ICU | 1 (0.4%) | 0 (0%) | 0 (0%) |  |  |
| Intermediate/Stepdown | 1 (0.4%) | 2 (0.4%) | 6 (1.0%) |  |  |
| Med-Surg ICU | 6 (2.5%) | 5 (0.9%) | 4 (0.7%) |  |  |
| MICU | 51 (21%) | 90 (17%) | 84 (14%) |  |  |
| Neuro ICU | 4 (1.6%) | 1 (0.2%) | 15 (2.5%) |  |  |
| PACU/Recovery | 1 (0.4%) | 2 (0.4%) | 2 (0.3%) |  |  |
| SICU | 96 (40%) | 225 (42%) | 252 (42%) |  |  |
| **Length of stay (days)** | 19.44 (11.79, 30.63) | 17.66 (11.25, 27.91) | 17.40 (10.60, 26.92) | 0.13 | 0.2 |
| **Heart rate** | 89.00 (77.00, 103.00) | 93.00 (80.00, 110.00) | 87.00 (75.00, 101.00) | <0.001 | <0.001 |
| Unknown | 5 | 19 | 11 |  |  |
| **Respiratory rate** | 20.00 (16.00, 24.00) | 20.00 (16.00, 25.00) | 18.00 (16.00, 22.00) | 0.021 | 0.053 |
| Unknown | 10 | 25 | 20 |  |  |
| **Temperature** | 36.61 (36.20, 37.17) | 36.44 (35.70, 36.89) | 36.44 (35.70, 37.00) | 0.045 | 0.093 |
| Unknown | 178 | 397 | 454 |  |  |
| **MAP** | 73.00 (66.00, 82.00) | 71.00 (64.00, 82.00) | 76.00 (67.00, 85.00) | <0.001 | 0.002 |
| Unknown | 56 | 106 | 100 |  |  |
| **APSIII** | 62.00 (50.00, 77.00) | 66.00 (49.00, 86.00) | 55.00 (41.00, 72.00) | <0.001 | <0.001 |
| **SAPSII** | 47.00 (37.00, 58.00) | 50.00 (40.00, 61.00) | 44.00 (36.00, 55.00) | <0.001 | <0.001 |
| **SOFA score** | 4.00 (3.00, 6.00) | 5.00 (3.00, 7.00) | 4.00 (2.00, 6.00) | <0.001 | <0.001 |
| **Congestive heart failure** | 75 (31%) | 142 (27%) | 175 (29%) | 0.5 | 0.6 |
| **Peripheral vascular disease** | 54 (22%) | 121 (23%) | 145 (24%) | 0.8 | 0.9 |
| **Cerebrovascular disease** | 37 (15%) | 74 (14%) | 114 (19%) | 0.073 | 0.14 |
| **Chronic pulmonary disease** | 73 (30%) | 135 (25%) | 162 (27%) | 0.4 | 0.6 |
| **Diabetes** | 79 (33%) | 154 (29%) | 196 (32%) | 0.4 | 0.6 |
| **Renal disease** | 67 (28%) | 118 (22%) | 123 (20%) | 0.090 | 0.2 |
| **Malignant cancer** | 20 (8.2%) | 46 (8.7%) | 46 (7.6%) | 0.8 | 0.9 |
| **Severe liver disease** | 34 (14%) | 54 (10%) | 66 (11%) | 0.3 | 0.4 |
| **Mechanical ventilation** | 242 (100%) | 527 (99%) | 602 (100%) | >0.9 | >0.9 |
| **RRT** | 116 (48%) | 184 (35%) | 147 (24%) | <0.001 | 0.002 |
| **In-hospital death** | 83 (34%) | 138 (26%) | 145 (24%) | 0.009 | 0.026 |

Data are presented as median (IQR) or n (%). P values were adjusted for multiple comparisons to obtain q values.

**Supplementary Table S14. Univariate analysis across PaO₂ trajectory classes**

| **Characteristic** | **1** | **2** | **3** | **p-value** | **q-value** |
| --- | --- | --- | --- | --- | --- |
| **Age** | 61.00 (47.00, 74.00) | 62.00 (51.00, 75.00) | 65.00 (53.00, 76.00) | 0.041 | 0.2 |
| **Gender** |  |  |  | 0.7 | 0.9 |
| F | 71 (42%) | 62 (38%) | 406 (39%) |  |  |
| M | 97 (58%) | 100 (62%) | 642 (61%) |  |  |
| **Race** |  |  |  | 0.071 | 0.2 |
| Asian | 2 (1.2%) | 8 (4.9%) | 21 (2.0%) |  |  |
| Black/African American | 17 (10%) | 7 (4.3%) | 68 (6.5%) |  |  |
| Hispanic/Latino | 4 (2.4%) | 6 (3.7%) | 44 (4.2%) |  |  |
| Native American | 0 (0%) | 1 (0.6%) | 2 (0.2%) |  |  |
| Other | 14 (8.3%) | 22 (14%) | 90 (8.6%) |  |  |
| White | 112 (67%) | 93 (57%) | 659 (63%) |  |  |
| Unknown | 19 (11%) | 25 (15%) | 164 (16%) |  |  |
| **ICU type** |  |  |  | <0.001 | 0.003 |
| Cardiac ICU | 44 (26%) | 42 (26%) | 444 (42%) |  |  |
| General ICU | 0 (0%) | 0 (0%) | 1 (<0.1%) |  |  |
| Intermediate/Stepdown | 1 (0.6%) | 1 (0.6%) | 7 (0.7%) |  |  |
| Med-Surg ICU | 1 (0.6%) | 2 (1.2%) | 12 (1.1%) |  |  |
| MICU | 26 (15%) | 14 (8.6%) | 185 (18%) |  |  |
| Neuro ICU | 4 (2.4%) | 4 (2.5%) | 12 (1.1%) |  |  |
| PACU/Recovery | 0 (0%) | 1 (0.6%) | 4 (0.4%) |  |  |
| SICU | 92 (55%) | 98 (60%) | 383 (37%) |  |  |
| **Length of stay (days)** | 19.04 (11.89, 28.30) | 18.15 (11.89, 28.38) | 17.30 (10.82, 27.91) | 0.5 | 0.8 |
| **Heart rate** | 92.00 (78.00, 110.00) | 89.00 (73.00, 106.00) | 89.00 (79.00, 104.00) | 0.4 | 0.6 |
| Unknown | 5 | 8 | 22 |  |  |
| **Respiratory rate** | 19.00 (16.00, 23.00) | 19.00 (16.00, 22.00) | 19.50 (16.00, 24.00) | 0.2 | 0.4 |
| Unknown | 5 | 7 | 43 |  |  |
| **Temperature** | 36.36 (35.21, 37.00) | 36.56 (35.33, 37.00) | 36.50 (35.80, 37.00) | 0.7 | 0.9 |
| Unknown | 138 | 129 | 762 |  |  |
| **MAP** | 73.00 (65.00, 81.00) | 76.00 (68.00, 86.00) | 73.00 (65.00, 84.00) | 0.11 | 0.3 |
| Unknown | 38 | 26 | 198 |  |  |
| **APSIII** | 65.00 (50.50, 84.00) | 56.00 (42.00, 75.00) | 59.00 (45.00, 78.00) | <0.001 | 0.003 |
| **SAPSII** | 50.00 (38.00, 59.00) | 42.00 (33.00, 55.00) | 47.00 (38.00, 58.00) | <0.001 | 0.003 |
| **SOFA score** | 4.00 (3.00, 6.00) | 3.00 (2.00, 5.00) | 4.00 (3.00, 6.00) | <0.001 | <0.001 |
| **Congestive heart failure** | 54 (32%) | 40 (25%) | 298 (28%) | 0.3 | 0.6 |
| **Peripheral vascular disease** | 31 (18%) | 33 (20%) | 256 (24%) | 0.2 | 0.4 |
| **Cerebrovascular disease** | 42 (25%) | 15 (9.3%) | 168 (16%) | <0.001 | 0.003 |
| **Chronic pulmonary disease** | 40 (24%) | 34 (21%) | 296 (28%) | 0.10 | 0.3 |
| **Diabetes** | 47 (28%) | 50 (31%) | 332 (32%) | 0.6 | 0.9 |
| **Renal disease** | 37 (22%) | 36 (22%) | 235 (22%) | >0.9 | >0.9 |
| **Malignant cancer** | 12 (7.1%) | 13 (8.0%) | 87 (8.3%) | >0.9 | >0.9 |
| **Severe liver disease** | 20 (12%) | 18 (11%) | 116 (11%) | >0.9 | >0.9 |
| **Mechanical ventilation** | 168 (100%) | 161 (99%) | 1,042 (99%) | 0.8 | >0.9 |
| **RRT** | 57 (34%) | 43 (27%) | 347 (33%) | 0.2 | 0.4 |
| **In-hospital death** | 50 (30%) | 41 (25%) | 275 (26%) | 0.6 | 0.8 |

Data are presented as median (IQR) or n (%). P values were adjusted for multiple comparisons to obtain q values.

**Supplementary Table S15**. Incremental Predictive Value of ABG Trajectory Features Beyond Reference Clinical Models

*Summary of discrimination, overall accuracy, and calibration improvement across landmark times*

| Landmark (day) | Cohort | Validation set | n | Delta ROC-AUC Mean (95% interval) | Delta PR-AUC Mean (95% interval) | Brier score reduction Mean (95% interval) | Calibration intercept improvement Mean (95% interval) | Calibration slope error improvement Mean (95% interval) |
| --- | --- | --- | --- | --- | --- | --- | --- | --- |
| 3 | MIMIC-IV | Training set | 25 | 0.009 (-0.002 to 0.023) | 0.023 (0.000 to 0.057) | 0.007 (-0.001 to 0.018) | 0.000 (0.000 to 0.000) | 0.275 (-0.214 to 1.270) |
| 3 | MIMIC-IV | Internal validation set | 25 | 0.002 (-0.031 to 0.023) | 0.011 (-0.020 to 0.060) | 0.005 (0.000 to 0.015) | -0.007 (-0.043 to 0.029) | 0.335 (-0.305 to 1.507) |
| 3 | eICU-CRD | External validation cohort | 25 | 0.036 (0.018 to 0.060) | 0.051 (0.025 to 0.090) | 0.008 (0.005 to 0.014) | 0.023 (-0.044 to 0.092) | 0.005 (-0.248 to 0.236) |
| 5 | MIMIC-IV | Training set | 25 | 0.018 (0.002 to 0.039) | 0.046 (0.010 to 0.080) | 0.013 (0.006 to 0.023) | 0.000 (0.000 to 0.000) | 0.568 (-0.060 to 1.549) |
| 5 | MIMIC-IV | Internal validation set | 25 | 0.009 (-0.038 to 0.041) | 0.028 (-0.029 to 0.065) | 0.010 (0.001 to 0.022) | -0.016 (-0.055 to 0.009) | 0.602 (-0.162 to 2.025) |
| 5 | eICU-CRD | External validation cohort | 25 | 0.044 (0.012 to 0.064) | 0.059 (0.007 to 0.094) | 0.010 (0.001 to 0.016) | -0.020 (-0.140 to 0.068) | 0.026 (-0.119 to 0.222) |
| 7 | MIMIC-IV | Training set | 25 | 0.035 (0.019 to 0.050) | 0.077 (0.050 to 0.105) | 0.019 (0.001 to 0.033) | 0.000 (0.000 to 0.000) | 0.534 (-0.675 to 1.860) |
| 7 | MIMIC-IV | Internal validation set | 25 | 0.030 (-0.021 to 0.063) | 0.057 (-0.016 to 0.115) | 0.015 (-0.005 to 0.032) | -0.018 (-0.080 to 0.026) | 0.505 (-0.861 to 1.914) |
| 7 | eICU-CRD | External validation cohort | 25 | 0.055 (0.026 to 0.081) | 0.066 (0.014 to 0.113) | 0.010 (0.003 to 0.020) | -0.042 (-0.214 to 0.120) | -0.115 (-0.297 to 0.172) |

Note: Values are means with 2.5th-97.5th percentile intervals. Positive values indicate incremental improvement of the combined model over the reference model. The clinically relevant reference model should include standard predictors such as age, SOFA, APACHE/APS, lactate, mechanical ventilation, vasopressor use, renal replacement therapy, and comorbidities. The combined model denotes the reference model plus ABG trajectory features and/or ABG summary statistics. ROC-AUC, area under the receiver operating characteristic curve; PR-AUC, area under the precision-recall curve; ABG, arterial blood gas.


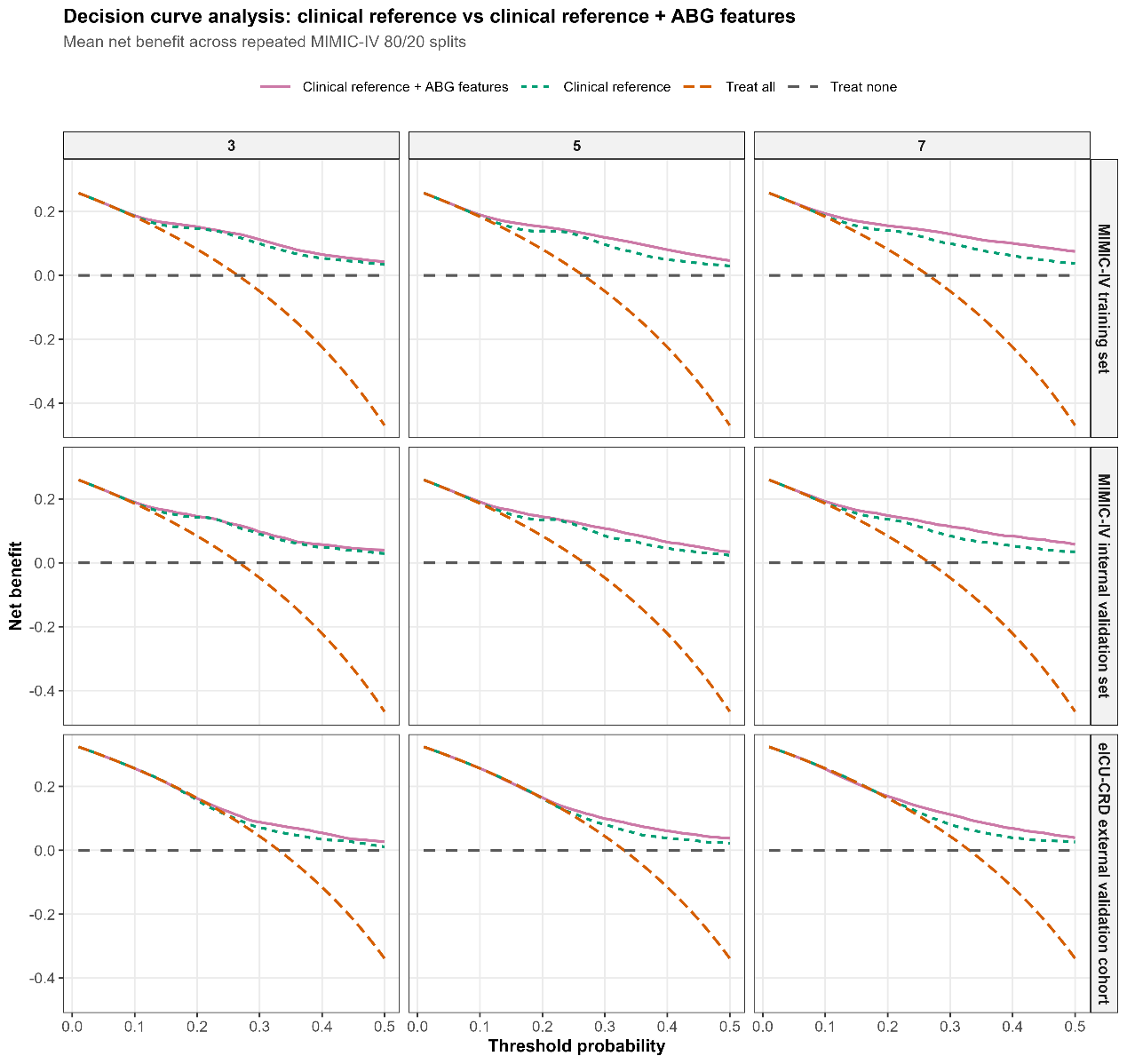


**Supplementary Figure S3**. Decision-curve analysis for dynamic prediction of post-landmark in-hospital mortality.

Decision curves were generated at day 3, day 5, and day 7 landmarks using only information available at or before each landmark. The clinical reference model was compared with the combined model incorporating ABG trajectory posterior probabilities and ABG summary features. Curves are shown for the MIMIC-IV training set, MIMIC-IV internal validation set, and eICU-CRD external validation cohort. “Treat all” and “treat none” are shown as reference strategies. The combined model provided higher net benefit across part of the threshold probability range, although the improvement was modest. ABG, arterial blood gas; eICU-CRD, eICU Collaborative Research Database.


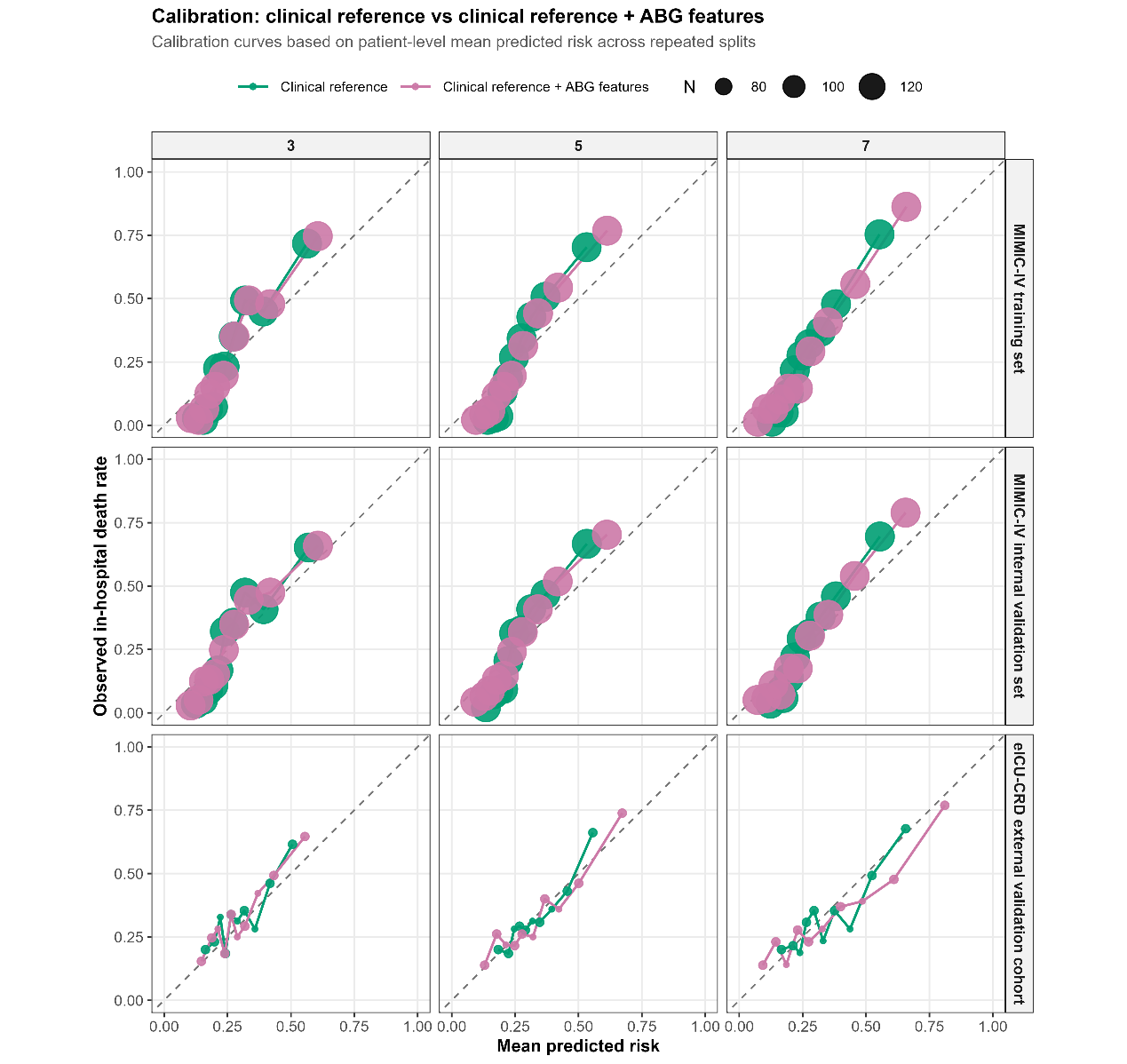


**Supplementary Figure S4. Calibration of dynamic models for post-landmark in-hospital mortality prediction.**

Calibration plots show observed versus predicted post-landmark in-hospital mortality at day 3, day 5, and day 7. Predictions used only clinical variables and ABG features available at or before each landmark. Patients who died or were discharged before the landmark were excluded. Points represent risk bins; point size indicates bin size, and the dashed line indicates perfect calibration. ABG, arterial blood gas.

**Supplementary Figure S5.** **Exploratory dynamic ROC-AUC for post-landmark incident septic shock prediction in MIMIC-IV**
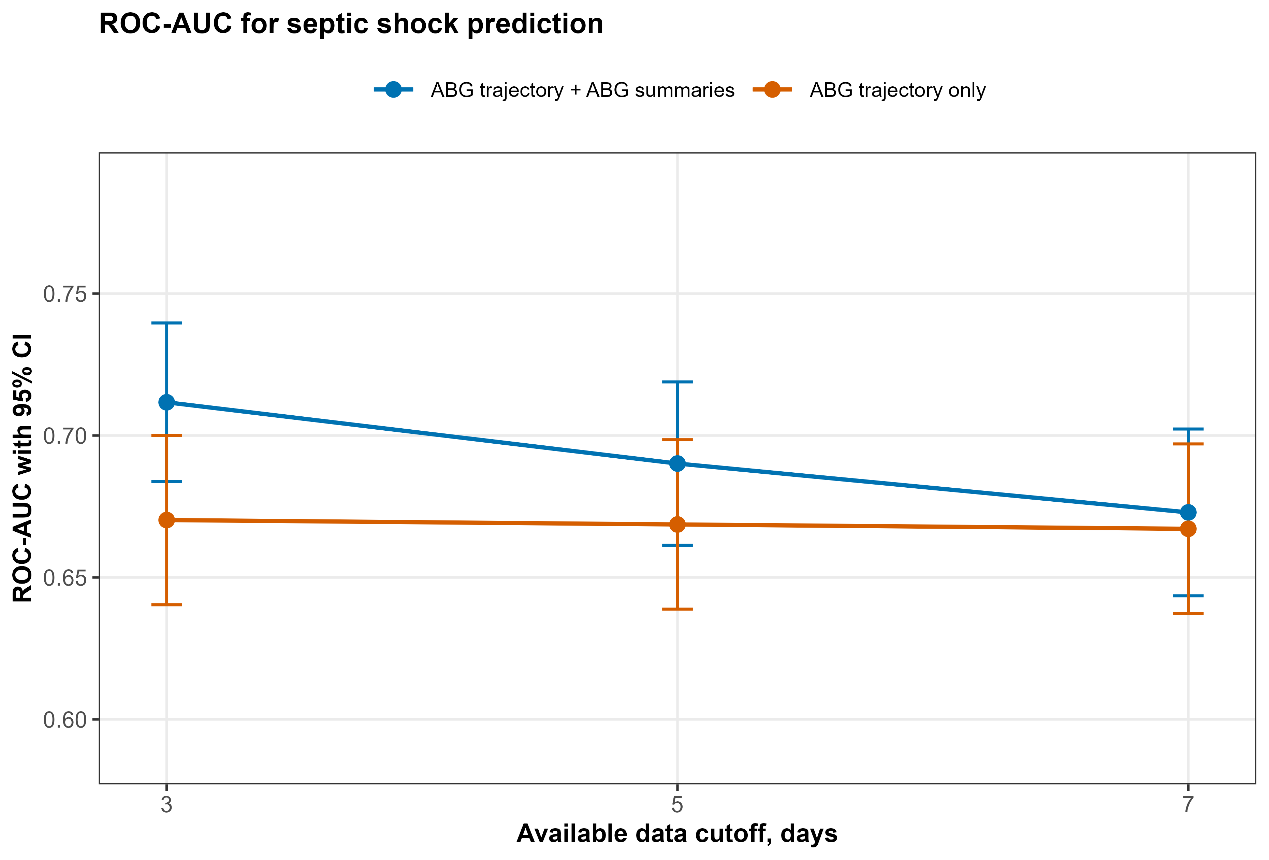


**Supplementary Figure S6. Exploratory dynamic ROC-AUC for post-landmark incident septic shock prediction in eICU-CRD**
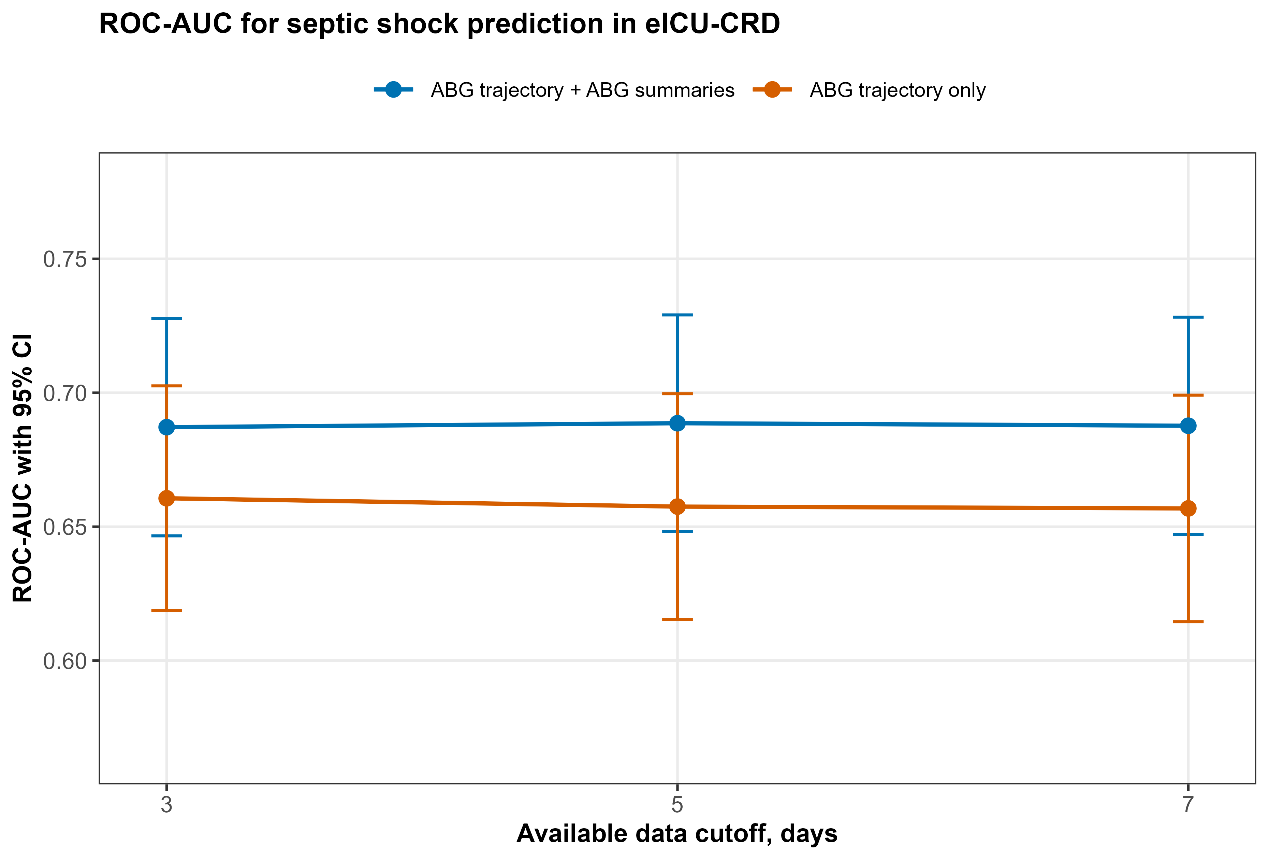


**Supplementary Table S16. Descriptive exploratory ROC-AUC of ABG trajectory models for incident septic shock in MIMIC-IV**

| **Model set** | **Landmark day** | **ROC-AUC** | **ROC-AUC (descriptive empirical interval)** |
| --- | --- | --- | --- |
| ABG trajectory + ABG summaries | 3 | 0.712 | 0.712 (0.684-0.740) |
| ABG trajectory only | 3 | 0.670 | 0.670 (0.640-0.700) |
| ABG trajectory + ABG summaries | 5 | 0.690 | 0.690 (0.661-0.719) |
| ABG trajectory only | 5 | 0.669 | 0.669 (0.639-0.699) |
| ABG trajectory + ABG summaries | 7 | 0.673 | 0.673 (0.644-0.702) |
| ABG trajectory only | 7 | 0.667 | 0.667 (0.637-0.697) |

Note. ROC-AUC, area under the receiver operating characteristic curve; ABG, arterial blood gas.

**Supplementary Table S17. Descriptive exploratory ROC-AUC of ABG trajectory models for incident septic shock in eICU-CRD**

| **Model set** | **Landmark day** | **ROC-AUC** | **ROC-AUC (descriptive empirical interval)** |
| --- | --- | --- | --- |
| ABG trajectory + ABG summaries | 3 | 0.687 | 0.687 (0.647-0.728) |
| ABG trajectory only | 3 | 0.661 | 0.661 (0.619-0.703) |
| ABG trajectory + ABG summaries | 5 | 0.689 | 0.689 (0.648-0.729) |
| ABG trajectory only | 5 | 0.657 | 0.657 (0.615-0.700) |
| ABG trajectory + ABG summaries | 7 | 0.688 | 0.688 (0.647-0.728) |
| ABG trajectory only | 7 | 0.657 | 0.657 (0.615-0.699) |

Note. ROC-AUC, area under the receiver operating characteristic curve; ABG, arterial blood gas.

**Supplementary Table S18. Baseline characteristics of patients included in the primary cohort and the sensitivity analysis cohort in MIMIC-IV**

| **Characteristic** | **Primary cohort (n = 1,378)** | **Sensitivity cohort (n = 3,772)** | **P value** | **\|SMD\|** |
| --- | --- | --- | --- | --- |
| ***Demographics and admission characteristics*** | | | | |
| Age, years | 64.00 [52.00, 75.00] | 65.00 [53.00, 76.00] | 0.264 | 0.043 |
| Hospital length of stay, days | 17.80 [11.03, 27.99] | 14.96 [9.01, 24.17] | <0.001 | 0.200 |
| Male sex | 839 (60.9) | 2,241 (59.4) | 0.340 | — |
| **Race** |  |  | 0.870 | — |
| White | 864 (62.7) | 2,292 (60.8) |  |  |
| Black/African American | 92 (6.7) | 296 (7.8) |  |  |
| Asian | 31 (2.2) | 101 (2.7) |  |  |
| Hispanic/Latino | 54 (3.9) | 132 (3.5) |  |  |
| Other/Unknown | 337 (24.5) | 951 (25.2) |  |  |
| **First ICU type** |  |  | <0.001 | — |
| Cardiac ICU | 530 (38.5) | 909 (24.1) |  |  |
| MICU/Medical ICU | 225 (16.3) | 1,521 (40.3) |  |  |
| SICU/Surgical ICU | 573 (41.6) | 1,262 (33.5) |  |  |
| Other ICU | 50 (3.6) | 80 (2.1) |  |  |
| ***Initial vital signs and severity scores*** | | | | |
| Heart rate, beats/min | 89.00 [78.00, 105.00] | 90.00 [77.00, 107.00] | 0.183 | 0.041 |
| Respiratory rate, breaths/min | 19.00 [16.00, 24.00] | 20.00 [16.00, 24.00] | <0.001 | 0.124 |
| Temperature, °C | 36.50 [35.80, 37.00] | 36.83 [36.39, 37.28] | <0.001 | 0.390 |
| Mean arterial pressure, mmHg | 74.00 [66.00, 84.00] | 74.00 [65.00, 85.00] | 0.513 | 0.044 |
| APS III | 60.00 [45.00, 78.00] | 60.00 [46.00, 79.00] | 0.837 | 0.005 |
| SAPS II | 47.00 [37.00, 58.00] | 46.00 [37.00, 57.00] | 0.323 | 0.029 |
| SOFA score | 4.00 [3.00, 6.00] | 4.00 [2.00, 6.00] | 0.001 | 0.107 |
| ***Comorbidities*** | | | | |
| Congestive heart failure | 392 (28.4) | 1,148 (30.4) | 0.168 | 0.044 |
| Peripheral vascular disease | 320 (23.2) | 562 (14.9) | <0.001 | 0.213 |
| Cerebrovascular disease | 225 (16.3) | 565 (15.0) | 0.234 | 0.037 |
| Chronic pulmonary disease | 370 (26.9) | 1,082 (28.7) | 0.195 | 0.041 |
| Diabetes mellitus | 429 (31.1) | 1,174 (31.1) | 0.996 | 0.000 |
| Renal disease | 308 (22.4) | 808 (21.4) | 0.473 | 0.022 |
| Malignant cancer | 112 (8.1) | 422 (11.2) | 0.001 | 0.104 |
| Severe liver disease | 154 (11.2) | 441 (11.7) | 0.608 | 0.016 |
| ***Organ support and outcome*** | | | | |
| Mechanical ventilation | 1,371 (99.5) | 3,475 (92.1) | <0.001 | 0.374 |
| Renal replacement therapy | 447 (32.4) | 532 (14.1) | <0.001 | 0.444 |
| Vasoactive/vasodilator therapy | 508 (36.9) | 2,789 (73.9) | <0.001 | 0.804 |
| In-hospital death | 366 (26.6) | 1,138 (30.2) | 0.012 | 0.080 |

**Note.** Values are median [IQR] or n (%). The primary cohort is the original analytical cohort; the sensitivity cohort used five ABG-related indicators over 7 days with 12-hour bins and at least three valid bins per indicator. P values were obtained from Wilcoxon rank-sum tests for continuous variables and chi-square or Fisher exact tests for categorical variables. |SMD| denotes absolute standardized mean difference. Race and ICU type were collapsed for concise presentation; their P values correspond to the overall categorical comparisons in the source analysis.

Supplementary Figure S7. Dynamic ROC-AUC for in-hospital mortality prediction in the five-variable sensitivity cohort


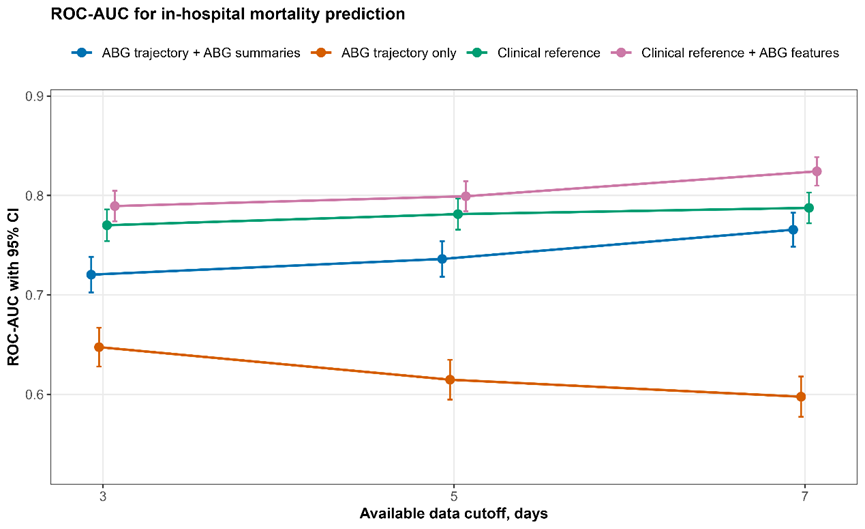


**Supplementary Table S19. Sensitivity analysis of dynamic prediction for in-hospital mortality using five core ABG variables**

| **Cutoff day** | **Model** | **ROC-AUC (95% CI)** | **CV AUC** |
| --- | --- | --- | --- |
| **Day 3** | ABG trajectory only | 0.648 (0.628-0.667) | 0.644 |
|  | ABG trajectory + ABG summaries | 0.720 (0.703-0.738) | 0.719 |
|  | Clinical reference | 0.770 (0.754-0.786) | 0.761 |
|  | Clinical reference + ABG features | 0.789 (0.774-0.805) | 0.779 |
| **Day 5** | ABG trajectory only | 0.615 (0.595-0.635) | 0.608 |
|  | ABG trajectory + ABG summaries | 0.736 (0.718-0.754) | 0.729 |
|  | Clinical reference | 0.781 (0.766-0.797) | 0.772 |
|  | Clinical reference + ABG features | 0.799 (0.784-0.814) | 0.787 |
| **Day 7** | ABG trajectory only | 0.598 (0.577-0.618) | 0.594 |
|  | ABG trajectory + ABG summaries | 0.766 (0.749-0.783) | 0.754 |
|  | Clinical reference | 0.788 (0.772-0.803) | 0.778 |
|  | Clinical reference + ABG features | 0.824 (0.810-0.839) | 0.802 |

Note: ABG, arterial blood gas; CI, confidence interval; CV, cross-validation. ROC-AUC values are shown with 95% confidence intervals. Event rate refers to in-hospital mortality.
